# Taxonomic studies on *Hygrophorus* in China: New species and revised taxonomy

**DOI:** 10.3897/mycokeys.134.186332

**Published:** 2026-06-17

**Authors:** Hong-Yan Huang, Wen-Hao Zhang, Tie-Zhi Liu, Guo-Li Zhang, Shu-Da Yang, Hai-Jiao Li, Gabriel Moreno, Li-Ping Tang

**Affiliations:** 1 College of Medicine, Lishui University, Lishui, 323000, Zhejiang, China School of Pharmaceutical Sciences and Yunnan Key Laboratory of Pharmacology for Natural Products, Kunming Medical University Kunming China; 2 School of Pharmaceutical Sciences and Yunnan Key Laboratory of Pharmacology for Natural Products, Kunming Medical University, Kunming, 650500, Yunnan, China College of Medicine, Lishui University Lishui China; 3 Yunnan College of Modern Biomedical Industry, Kunming, 650500, Yunnan, China Facultad de Ciencias, University of Alcalá Madrid Spain; 4 College of Life Sciences, Chifeng University, Chifeng, 024000, Inner Mongolia, China National Institute of Occupational Health and Poison Control, Chinese Center for Disease Control and Prevention Beijing China; 5 National Institute of Occupational Health and Poison Control, Chinese Center for Disease Control and Prevention, 100050, Beijing, China College of Life Sciences, Chifeng University Chifeng China; 6 Facultad de Ciencias, University of Alcalá, Alcalá de Henares, 28805, Madrid, Spain Yunnan College of Modern Biomedical Industry Kunming China

**Keywords:** Edible mushroom, Hygrophoraceae, molecular phylogeny, new taxa, species complexes

## Abstract

A taxonomic revision of the genus *Hygrophorus* in China was conducted using integrated morphological and molecular phylogenetic analyses, based on sequences of the internal transcribed spacer (ITS) region, the large subunit nuclear ribosomal RNA (nrLSU) and the translation elongation factor 1-alpha (*tef1-α*) gene. Our study confirms the recognition of five new species as *H. atropurpureus* and *H. ochraceodiscus* in sect. *Hygrophorus*; *H. paulus* and *H. shennongjiaensis* in sect. *Pudorini*; and *H. aurantiophyllus* in sect. *Vividi*. Phylogenetic analysis, based on ITS data and the concatenated ITS + nrLSU + *tef1-α* dataset, demonstrated that *H. atropurpureus* groups with H. aff. atropurpureus; *H. ochraceodiscus* is sister to *H. brunneodiscus*; *H. aurantiophyllus* forms a clade with *H. vividus* and an undescribed taxon; and *H. paulus* and *H. shennongjiaensis* group together with *H. nemoreus*. Detailed morphological descriptions, ecological notes and updated geographical distribution records are provided for these new taxa as well as for several previously described species. These findings bring the total number of confirmed *Hygrophorus* taxa in China to 63, establishing a more robust framework for understanding the diversity of this genus in the region.

## Introduction

The genus *Hygrophorus* Fr., commonly known as waxcaps, comprises ectomycorrhizal basidiomycetes, characterised by mostly viscid to glutinous basidiomata, adnate to decurrent lamellae with divergent or bilateral hymenophoral trama and the presence of clamp connections. These fungi are widely distributed across temperate regions of the Northern Hemisphere, particularly in high-latitude or high-altitude zones, such as North America, northern Europe and south-western and north-eastern China (Hesler and Smith 1963; Candusso 1997; Huang et al. 2018, 2021a, 2021b, 2022a, 2022b; Wang et al. 2023; Bellanger et al. 2025; Papetti 2025). Most species exhibit restricted geographical ranges, although at least 19 are considered transcontinental in distribution (Lodge et al. 2014; Bellanger et al. 2021; Huang et al. 2021a, 2021b, 2022a, 2022b; Wang et al. 2023). Ecologically, most form ectomycorrhizal associations with conifers (e.g. *Abies*, *Larix*, *Picea* and *Pinus*) or broadleaf trees (e.g. *Betula*, *Fagus* and *Quercus*). A few species, such as *H. olivaceoalbus* (Fr.) Fr. and *H. penarius* Fr., may exhibit facultative parasitic behaviour (Marino 2008; Agerer 2012).

The infrageneric classification of *Hygrophorus* has been progressively clarified through molecular phylogenetics. Following the phylogenetic framework proposed by Lodge et al. (2014), the genus is divided into three subgenera: *Camarophyllus* Fr., *Colorati* (Bataille) E. Larss. and *Hygrophorus*. The subgenus *Hygrophorus*, which is monophyletic in nrLSU analyses, comprises three sections: sect. *Discoidei* (Bataille) Konrad & Maubl., sect. *Hygrophorus* and sect. *Picearum* E. Larss. (Lodge et al. 2014). In contrast, the subgenus *Colorati* is paraphyletic and represents the most species-rich lineage. Lodge et al. (2014) initially divided this subgenus into three sections — sect. *Aurei* (Bataille) E. Larss., sect. *Olivaceoumbrini* (Bataille) Konrad & Maubl. and sect. *Pudorini* (Bataille) Konrad & Maubl. — based primarily on ITS sequences, supplemented by limited nrLSU, SSU, and *rpb2* sequences. This framework was subsequently refined by Bellanger et al. (2021), who split sect. *Olivaceoumbrini* s.l. into five sections, based on ITS sequences: sect. *Fuscocinerei* Bon ex Bellanger, P.-A. Moreau & E. Larss., sect. *Limacini* P.-A. Moreau, Bellanger, Loizides & E. Larss., sect. *Nudolidi* Bellanger & Lebeuf, sect. *Olivaceoumbrini* s.s. and sect. *Tephroleuci* (Bataille) Candusso. More recently, Wang et al. (2023) adopted the subdivision of Lodge et al. (2014) using a multi-locus dataset and proposed a new section, i.e. sect. *Vividi* C.Q. Wang, Ming Zhang & T.H. Li, further enriching the taxonomic structure of the genus.

Given this background of ongoing taxonomic refinement, China has emerged as a region of remarkable diversity for *Hygrophorus*. Several species, such as *H. annulatus* C.Q. Wang & T.H. Li, *H. hedrychii* (Velen.) K. Kult, *H. lucorum* Kalchbr., *H. russula* (Schaeff. ex Fr.) Bataille complex and *H. xiangjun* H.Y. Huang & L.P. Tang, are locally marketed as edible fungi (Wang and Li 2020; Wang et al. 2020, 2021, 2023; Huang et al. 2021a, 2022a, 2022b). To date, at least 99 species have been reported from China. Amongst these, 58 taxa have been confirmed, including 46 originally described from China (Huang et al. 2018, 2021a, 2021b, 2022a, 2022b; Wang and Li 2020; Wang et al. 2020, 2021, 2023; Zhang et al. 2023, 2024), while 41 previously recorded taxa require further verification. Notably, one species originally reported from China, *H. robustus* F.Q. Yu, has been reclassified into the genus *Tricholoma*, as *T. aurantiophyllum* L.P. Tang, H.Y. Huang, F.Q. Yu & R. Wang (Yu et al. 2007; Huang et al. 2024), highlighting the need for continued taxonomic scrutiny.

Recent studies have consistently revealed that the diversity of *Hygrophorus* in China far exceeds earlier estimations (Huang et al. 2018, 2021a, 2021b, 2022a, 2022b; Wang and Li 2020; Wang et al. 2020, 2021, 2023; Zhang et al. 2023, 2024). Wang et al. (2023) conducted a multi-locus (ITS-nrLSU-*rpb2*-*tef1-α*) phylogenetic analysis that identified 62 taxa (including five insufficiently known species) of *Hygrophorus* in China. In the present study, we combined morphological examination with molecular phylogenetic analyses, based on a multi-locus dataset (ITS, nrLSU and *tef1-α*) from collections in China, Spain Sweden and the United Kingdom. Our objectives were: 1) to report new findings and further enrich the known species diversity of *Hygrophorus* in China; 2) to evaluate the phylogeny of the genus with the inclusion of newly-sequenced specimens; and 3) to provide supplementary details on the morphology, ecology and distribution of ten previously reported Chinese taxa for which we had relatively in-depth studies, allowing us to meaningfully update their taxonomy.

## Materials and methods

### Sampling and morphological studies

Most of the fresh fruiting bodies from China in this study were collected from coniferous forests or mixed coniferous and broadleaf forests during the rainy season (July–October). Samples were photographed in situ using a Panasonic DMC LX10 camera. Fresh macroscopic characteristics (size, colour, shape, context discolouration upon injury, basal mycelium colour etc.) and collection details (geographic location, altitude, associated tree species and habitat conditions) were recorded. Specimen processing consisted of two parts: (1) for molecular materials, a small portion of fruiting body tissue was excised, wrapped in tissue paper and placed in a resealable bag containing indicating silica gel for drying at room temperature; the silica gel was replaced promptly until complete dryness was achieved; (2) for morphological specimens, the remaining fruiting body was dried using an electric dryer at 50–60 °C to prepare preserved dried specimens.

*Hygrophorus* collections from China, Spain, Sweden and the United Kingdom were included in this study. Voucher specimens and field records were deposited in the following Herbaria: the University of Alcalá (AH), Spain; the Herbarium of Cryptogams, Kunming Institute of Botany, Chinese Academy of Sciences (HKAS), Kunming, China; Mycological Herbarium, Institute of Microbiology, Chinese Academy of Sciences (HMAS), Beijing, China; the Mycological Herbarium of Kunming Medical University (MHKMU), Kunming, China; and the Herbarium of the Royal Botanic Garden, Edinburgh (E), UK.

Macro-morphological descriptions were based on the field notes and images of fresh basidiomata. Colour codes (e.g. 1A2–3) follow Kornerup and Wanscher (1981). Micro-morphological characteristics were examined using a Leica DM2500 microscope. Sections of dried specimens were prepared and mounted in 5% potassium hydroxide (KOH), with 1% Congo Red solution (w/v) when necessary for staining. Basidiospore amyloidity was tested using Melzer’s reagent. For detailed surface observation, basidiospores were examined with a ZEISS Sigma 300 scanning electron microscope (SEM) at 8.00 kV. These procedures generally followed those described by Huang et al. (2018, 2020).

In the basidiospore descriptions, the abbreviations [n/m/p] indicate n basidiospores were measured from m basidiomata of p collections. Basidiospores dimensions are presented as (a) b–c (d), where the range of b–c contains at least 90% of the measured values and ‘a’ and ‘d’ represent the extreme values. The notation Q denotes the length/width ratio of basidiospores in profile view and Q_m_ refers to the mean Q of all basidiospores ± sample standard deviation.

### DNA extraction, PCR amplification and sequencing

Total genomic DNA was extracted from 10–20 mg of dried basidioma tissue from each voucher specimen using a modified CTAB method (Doyle and Doyle 1987). ITS, nrLSU and *tef1-α* were amplified via a polymerase chain reaction (PCR) using primer pairs ITS5/ITS4 (White et al. 1990), LR0R/LR5 (Vilgalys and Hester 1990) and EF1-983F/1567R (Rehner and Buckley 2005), respectively. Each 25 μl PCR mixture contained: 2.5 μl 10 × amplification buffer (with MgCl_2_), 0.5 μl dNTP (200 μM), 0.2 μl Taq DNA polymerase (5 U/μl), 1 μl each primer (10 μM), 1 μl DNA template and 18.8 μl sterile water. Thermal cycling conditions followed those described by Tang et al. (2017). PCR products were separated by electrophoresis on 1% agarose gels to confirm amplification and successful products were sequenced on an ABI 3730 DNA Analyzer (Sangon, Shanghai, China) using the same primers as for amplification.

### Sequence alignment and phylogenetic analyses

Newly-generated sequences were assembled and edited using SeqMan (DNASTAR Lasergene 9) and deposited in GenBank (http://www.ncbi.nlm.nih.gov; see Table 1 for accession numbers). Multiple sequence alignments were initially generated with MAFFT v.7.03 (Katoh and Standley 2013) and subsequently adjusted manually in BioEdit v.7.0.9 (Hall 1999). Phylogenetic analyses were performed separately for the ITS data and a concatenated dataset comprising ITS, nrLSU and *tef1-α*, using both Maximum Likelihood (ML) and Bayesian Inference (BI) methods.

**Table 1.** Sequences used/produced in the present study. New species are shown in bold; * refers to type material (holotype) and — refers to the missing data.

Taxon	Voucher	Locality	GenBank accession no.			References
			ITS	nrLSU	*tef1-α*	
* Chrysomphalina aurantiaca*	UBC:F34015	Canada	ON738529	—	—	Berbee et al. unpublished
* C. aurantiaca*	iNAT:9391403	USA	OM212944	—	—	Miller et al. unpublished
* C. grossula*	OSC 113667	USA	EU644703	EU652372	—	Gordon unpublished
* C. grossula*	OSC 113683	USA	EU644704	EU652373	—	Gordon unpublished
* Hygrophorus abieticola*	HKAS 63388	Heilongjiang, China	PX868418	PX868322	PX907451	This study
* H. abieticola*	GDGM44322	Sichuan, China	OP547627	OP558617	OR575224	Wang et al. (2023)
* H. adiaphorus*	MICH10867*	USA	MT981606	—	—	Bellanger et al. (2021)
* H. aesculeticola*	MES-3095	USA	MK020098	—	—	Smith unpublished
H. aff. alboflavescens	HMAS 253522	Tibet, China	PX868419	PX868323	PX907452	This study
H. aff. alpinus	MHKMU HHY-1171-1	Hunan, China	PX868420	PX868324	PX907453	This study
H. aff. alpinus	MHKMU HHY-1171-2	Hunan, China	PX868421	PX868325	PX907454	This study
H. aff. alpinus	MHKMU HHY-1171-3	Hunan, China	PX868422	PX868326	PX907455	This study
H. aff. alpinus	MHKMU HHY-1173-1	Hunan, China	PX868423	PX868327	—	This study
H. aff. alpinus	MHKMU HHY-1173-2	Hunan, China	PX868424	PX868328	—	This study
H. aff. alpinus	MHKMU HHY-1173-3	Hunan, China	PX868425	PX868329	—	This study
H. aff. atropurpureus	MHKMU PYJ-508	Yunnan, China	PX868426	PX868330	PX907456	This study
H. aff. atropurpureus	XHW7387 (HKAS131116)	China	OP547778	OP558766	—	Wang et al. (2023)
H. aff. brunneodiscus	HKAS 61774	Jilin, China	PX868427	PX868331	—	This study
H. aff. brunneodiscus	HMAS 267828	Inner Mongolia, China	PX868428	PX868332	PX907457	This study
H. aff. brunneodiscus	HMAS 290355	Jilin, China	PX868429	PX868333	—	This study
H. aff. brunneodiscus	HMAS 290369	Jilin, China	—	PX868334	—	This study
H. aff. brunneodiscus	HMAS 290396	Jilin, China	—	PX868335	—	This study
H. aff. chrysodon	Montri-249	Switzerland	MK028419	—	—	Hofstetter et al. (2019)
H. aff. hedrychii	HMAS 250528	Heilongjiang, China	PX868430	—	—	This study
H. aff. yukishiro	E00905352	England	PX868431	—	—	This study
H. aff. yukishiro	E00905353	England	PX868432	—	—	This study
* H. agathosmoides*	HMAS 281303	Inner Mongolia, China	MZ605814	ON764316	MZ614633	Huang et al. (2022b)
* H. agathosmoides* f. *trabzonensis*	KATO Fungi 3604	Turkey	MG888785	MG888786	—	Bellanger et al. (2021)
* H. agathosmus*	EL398-17*	Sweden	MH656445	—	—	Larsson et al. (2018)
* H. albofloccosus*	UCSCF2171*	USA	MT981691	—	—	Bellanger et al. (2021)
* H. albofloccosus*	GDGM70044	USA	OP547639	OP558630	OR575228	Wang et al. (2023)
* H. albofloccosus*	GDGM70063	USA	OP547642	OP558633	OR575229	Wang et al. (2023)
* H. alboflavescens*	LAH35243*	Pakistan	MK066236	MK066240	—	Naseer et al. (2019)
* H. alboflavescens*	HMJAU61972	Jilin, China	OL989473	—	—	Dong and Bau unpublished
* H. alboflavescens*	GDGM82808	Sichuan, China	OP547675	OP558668	OR575227	Wang et al. (2023)
* H. alpinus*	MHKMU ZWH-463-2*	Yunnan, China	MW762963	MW762753	OK063788	Huang et al. (2022a)
* H. alpinus*	MHKMU ZWH-463-3	Yunnan, China	MW762964	MW762754	OK063789	Huang et al. (2022a)
* H. annulatus*	GDGM45124*	Sichuan, China	MT758324	OQ867282	OR575237	Wang et al. (2021)
* H. annulatus*	MHKMU HHY-818	Yunnan, China	MZ605819	MZ605781	MZ614635	Huang et al. (2022b)
* H. arbustivus*	E00905354	England	PX908898	—	—	This study
* H. armeniacus*	GDGM82364*	Yunnan, China	OP547665	OP558658	—	Wang et al. (2023)
* H. atrofuscus*	MHKMU HT-541	Yunnan, China	ON764314	ON764317	ON787968	Huang et al. (2022b)
* H. atropurpureus*	HKAS 49694	Yunnan, China	PX868433	—	—	This study
* H. atropurpureus*	MHKMU TLP-3516*	Yunnan, China	PX868434	PX868336	PX907458	This study
* H. aurantioluteus*	SAAS191*	Sichuan, China	OP547759	OP558743	—	Wang et al. (2023)
* H. aurantioluteus*	HKAS 112566	Yunnan, China	MW762877	MW763002	MW773441	He and Yang (2021)
* H. aurantiophyllus*	MHKMU HHY-1004-1*	Yunnan, China	PX868435	PX868337	PX907459	This study
* H. aurantiophyllus*	MHKMU HHY-1004-2	Yunnan, China	PX868436	PX868338	PX907460	This study
* H. aurantiosquamosus*	HKAS 80742*	Tibet, China	OK104067	OK104061	OK999992	Huang et al. (2021b)
* H. aurantiosquamosus*	HKAS 82501	Tibet, China	MW616463	MW600482	MW656462	He and Yang (2021)
* H. aurorae*	MQ22-HRL3616	Canada	OQ740673	—	—	Landry & Lebeuf unpublished
* H. bakerensis*	DAVFP26678	Canada	JF899556	—	—	Guichon et al. unpublished
* H. betulae*	KM850911*	Finland	MK123936	—	—	Larsson and Bendiksen (2020)
* H. boyeri*	QFB 29507*	Canada	MG882094	—	—	Moreau et al. (2018)
* H. brunneiceps*	HMAS 280906	Tibet, China	MZ605842	MZ605799	MZ614643	Huang et al. (2022b)
* H. brunneiceps*	HMAS 254315	Tibet, China	MZ605838	MZ605795	MZ614641	Huang et al. (2022b)
* H. brunneiceps*	HMAS 254317	Tibet, China	MZ605839	MZ605796	MZ614642	Huang et al. (2022b)
* H. brunneodiscus*	GDGM76934	Hunan, China	MT093605	MT093621	OR575246	Wang et al. (2020)
* H. brunneodiscus*	GDGM73213*	Hunan, China	MN378318	MT093623	—	Wang et al. (2020)
* H. brunneoloaurantiacus*	MHKMU ZGL-113	Yunnan, China	PX868437	PX868339	PX907461	This study
* H. brunneoloaurantiacus*	HKAS 69410	Yunnan, China	PX868438	PX868340	PX907462	This study
* H. brunneoloaurantiacus*	MHKMU TLP-1908	Yunnan, China	PX868439	PX868341	—	This study
* H. brunneoloaurantiacus*	MHKMU ZJ-122	Yunnan, China	PX868440	PX868342	PX907463	This study
* H. brunneoloaurantiacus*	MHKMU YSD-121	Yunnan, China	PX868441	PX868343	PX907464	This study
* H. brunneololamellatus*	GDGM84600*	Sichuan, China	OP547716	OP558708	OR575253	Wang et al. (2023)
* H. brunneolus*	SAAS510	Sichuan, China	OP547761	OP558745	—	Wang et al. (2023)
* H. brunneolus*	SAAS617*	Sichuan, China	OP547762	OP558746	—	Wang et al. (2023)
* H. brunnescens*	GDGM84456*	Sichuan, China	OP547694	OP558686	OR575248	Wang et al. (2023)
* H. camarophyllus*	CR11	Canada	KP406544	—	—	Kranabetter et al. unpublished
* H. canadensis*	DAOM HRL2344*	Canada	NR172980	—	—	Bellanger et al. (2021)
* H. capreolarius*	EMB_2171/14	Italy	MF399420	—	—	Peintner et al. unpublished
* H. chrysaspis*	TENN:070772	USA	MF686502	—	—	Matheny & Sanchez-Garcia unpublished
* H. chrysodon*	E00905347	Sweden	OK104068	—	—	Huang et al. (2021b)
* H. chrysodon*	Hal-BP-113	Italy	MT594510	—	—	Leonardi et al. (2020)
* H. chrysodon*	E00905348	USA	OK104069	OK104062	—	Huang et al. (2021b)
H. cf. eburneus	JLF7239	USA	MK737049	—	—	Frank unpublished
H. cf. exiguus	GDGM44612	Jilin, China	OP547635	OP558625	—	Wang et al. (2023)
H. cf. mesotephrus	TENN 71861	USA	MG773826	MT237450	—	Matheny et al. unpublished
H. cf. purpurascens	Mushroom Observer 276944	USA	MN190180	—	—	Clements unpublished
H. cf. pusillus	GDGM70043	USA	OP547638	OP558629	OR575250	Wang et al. (2023)
H. cf. subalpinus	TRTC156469	Canada	JN021041	—	—	Dentinger et al. (2011)
* H. chuxiongensis*	GDGM83746*	China	OP547689	OP558681	OR575252	Wang et al. (2023)
* H. cinnamoneus*	GDGM84654	Sichuan, China	OP547721	OP558713	OR575255	Wang et al. (2023)
* H. cinnamoneus*	GDGM84699*	Sichuan, China	OP547726	OP558718	OR575256	Wang et al. (2023)
* H. cossus*	Sowerby 1794	United Kingdom	AY242852	—	—	Larsson and Jacobsson (2004)
* H. deliciosus*	GDGM79208*	Yunnan, China	MT363808	OQ867281	OR575258	Wang and Li (2020)
* H. deliciosus*	MHKMU HHY-1006	Yunnan, China	MW290174	MW290237	MW928553	Huang et al. (2021a)
* H. discoideus*	TU106301	Estonia	UDB015804	—	—	https://unite.ut.ee/
* H. discoxanthus*	SJ97044	Sweden	AY242853	—	—	Larsson and Jacobsson (2004)
* H. eburneus*	BP98806	Hungary	MK088116	—	—	Zajta et al. (2019)
* H. eburneus*	src592	USA	EF559291	—	—	Smith et al. (2007)
* H. esculentus*	MHKMU PYJ-321*	Yunnan, China	MW762967	MW762757	OK063792	Huang et al. (2022a)
* H. esculentus*	MHKMU MM-718	Yunnan, China	MW762966	MW762756	OK063791	Huang et al. (2022a)
* H. erubescens* var. *gracilis*	TENN:012497	USA	HQ185700	—	—	Matheny & Wolfenbarger unpublished
* H. exiguus*	TURA190791*	Finland	KJ720198	—	—	Larsson et al. (2014)
* H. flavoalbus*	MHKMU HHY-186	Yunnan, China	PX868442	PX868344	PX907465	This study
* H. flavoalbus*	MHKMU HHY-943	Yunnan, China	PX868443	PX868345	PX907466	This study
* H. flavoalbus*	MHKMU HHY-944	Yunnan, China	PX868444	PX868346	PX907467	This study
* H. flavodiscus*	GDGM70070	USA	OP547646	OP558637	—	Wang et al. (2023)
* H. flavodiscus*	DSH101009.2	USA	HM020691	HM026550	—	Seitzman & Hibbett unpublished
* H. fragilipurpurascens*	GDGM44332*	Sichuan, China	OP547632	OP558622	OR575233	Wang et al. (2023)
* H. fragilipurpurascens*	HKAS 71779	Yunnan, China	PX868445	—	—	This study
* H. fragilipurpurascens*	HKAS 72568	Yunnan, China	PX868446	—	—	This study
* H. fragilipurpurascens*	MHKMU HT-472	Yunnan, China	PX868447	PX868347	PX907468	This study
* H. fragrans*	IB19950382	USA	MF399446	—	—	Peintner et al. unpublished
* H. fuligineus*	GDGM70057	USA	OP547641	OP558632	OR575263	Wang et al. (2023)
* H. fuligineus*	GDGM70064	USA	OP547643	OP558634	OR575264	Wang et al. (2023)
* H. fuligineus*	HRL1034	Canada	MG882093	—	—	Moreau et al. (2018)
* H. fuscoalboides*	MICH5572/AHS46726*	USA	MT981636	—	—	Bellanger et al. (2021)
* H. fuscodiscus*	GDGM84543	Sichuan, China	OP547708	OP558700	OR575265	Wang et al. (2023)
* H. fuscodiscus*	GDGM84677*	Sichuan, China	OP547723	OP558715	OR575266	Wang et al. (2023)
* H. fuscopapillatus*	GDGM44412*	Sichuan, China	MN378337	MT093625	OR575267	Wang et al. (2020)
* H. fuscopapillatus*	MHKMU HHY-912	Yunnan, China	OK011525	OK001710	OK063785	Huang et al. (2022b)
* H. fuscopapillatus*	MHKMU TLP-1784	Yunnan, China	PX868448	PX868348	—	This study
* H. fuscopapillatus*	MHKMU ZJ-29	Yunnan, China	PX868449	PX868349	PX907469	This study
* H. fuscopapillatus*	MHKMU YSD-87	Yunnan, China	PX868450	PX868350	PX907470	This study
* H. fuscopapillatus*	MHKMU ZGL-112	Yunnan, China	PX868451	PX868351	PX907471	This study
* H. fuscopapillatus*	HKAS 54976	Yunnan, China	PX868452	PX868352	PX907472	This study
* H. fuscopapillatus*	Q. Zhao 388	Yunnan, China	—	PX868353	—	This study
* H. fuscopapillatus*	HKAS 63564	Yunnan, China	PX868453	PX868354	PX907473	This study
* H. fuscopapillatus*	MHKMU WJ-25	Yunnan, China	PX868454	PX868355	—	This study
* H. fuscopapillatus*	MHKMU HHY-161	Yunnan, China	OK104070	OK104063	OK999993	Huang et al. (2021b)
* H. fuscopapillatus*	MHKMU YSD-546	Yunnan, China	PX868455	PX868356	PX907474	This study
* H. fuscopapillatus*	MHKMU TLP-2579	Yunnan, China	PX868456	PX868357	—	This study
* H. fuscopapillatus*	MHKMU TLP-2580	Yunnan, China	—	PX868358	PX907475	This study
* H. fuscopapillatus*	MHKMU YSD-16	Yunnan, China	PX868457	—	—	This study
* H. fuscopapillatus*	MHKMU MM-251	Yunnan, China	PX868458	PX868359	—	This study
* H. fuscopapillatus*	MHKMU MM-279	Yunnan, China	PX868459	PX868360	—	This study
* H. fuscopapillatus*	MHKMU TLP-2694	Yunnan, China	PX868460	PX868361	—	This study
* H. fuscopapillatus*	MHKMU HHY-309	Yunnan, China	PX868461	PX868362	—	This study
* H. fuscopapillatus*	MHKMU ZWH-206	Yunnan, China	PX868462	PX868363	—	This study
* H. fuscopapillatus*	MHKMU MM-795	Yunnan, China	PX868463	PX868364	PX907476	This study
* H. fuscopapillatus*	MHKMU TLP-3298	Yunnan, China	—	PX868365	PX907477	This study
* H. fuscopapillatus*	MHKMU TLP-3463	Yunnan, China	PX868464	PX868366	PX907478	This study
* H. fuscopapillatus*	MHKMU NX-157	Yunnan, China	PX868465	—	—	This study
* H. fuscopapillatus*	MHKMU HT-496	Yunnan, China	PX868466	PX868367	PX907479	This study
* H. fuscopapillatus*	MHKMU HHY-856	Yunnan, China	PX868467	PX868368	PX907480	This study
* H. fuscopapillatus*	MHKMU HHY-891	Yunnan, China	PX868468	PX868369	—	This study
* H. fuscopapillatus*	MHKMU PYJ-400	Yunnan, China	PX868469	PX868370	PX907481	This study
* H. gliocyclus*	MHKMU HHY-877	Yunnan, China	MW762984	MW762772	OK063799	Huang et al. (2022a)
* H. gliocyclus*	MHKMU HHY-913	Yunnan, China	MW762985	MW762773	OK063800	Huang et al. (2022a)
* H. gliocyclus*	181-324	USA	MH038136	—	—	Hart et al. (2018)
* H. glutiniceps*	MHKMU HHY-797	Hainan, China	OK011526	OK001711	OK063786	Huang et al. (2022b)
* H. glutinosus*	TENN061762	USA	MN243161	—	—	Bellanger et al. (2021)
* H. glutinifer*	E00218159	Scotland	MZ605844	MZ605800	—	Huang et al. (2022b)
* H. goetzei*	iNAT:125388551	USA	OP751547	—	—	Singer unpublished
* H. griseodiscus*	GDGM84644	Sichuan, China	OP547720	OP558712	OR575271	Wang et al. (2023)
* H. griseodiscus*	SAAS462*	Sichuan, China	MN378338	MT093624	OR575272	Wang et al. (2020)
* H. habaensis*	MHKMU ZWH-513-1*	Yunnan, China	MZ605845	MZ605801	MZ614644	Huang et al. (2022b)
* H. habaensis*	MHKMU ZWH-513-2	Yunnan, China	MZ605846	MZ605802	MZ614645	Huang et al. (2022b)
* H. hedrychii*	GDGM84408	Sichuan, China	OP547691	OP558683	OR575274	Wang et al. (2023)
* H. hedrychii*	HMAS 290140	Inner Mongolia, China	OK011527	OK001712	OK063787	Huang et al. (2022b)
* H. hedrychii*	E00905313	England	PX868470	—	—	This study
* H. hedrychii*	E00905314	Scotland	PX868471	PX868371	—	This study
* H. hedrychii*	E00905335	Sweden	PX868472	PX868372	—	This study
* H. hedrychii*	CFSZ 21431	Inner Mongolia, China	PX868473	PX868373	PX907482	This study
* H. hedrychii*	HMAS 250535	Heilongjiang, China	PX868474	PX868374	—	This study
* H. hedrychii*	HMAS 255531	Inner Mongolia, China	PX868475	PX868375	—	This study
* H. hedrychii*	HMAS 281316	Inner Mongolia, China	PX868476	—	PX907483	This study
* H. hyacinthinus*	JMM10092801/LIP0401691*	France	MT845204	—	—	Bellanger et al. (2021)
* H. hypothejus*	KUN-HKAS 56550	Germany	MW762987	MW762775	—	Huang et al. (2022a)
* H. inocybiformis*	SJ94011	Sweden	UDB000565	—	—	https://unite.ut.ee/
* H. korhonenii*	H6035782*	Finland	MT981641	—	—	Bellanger et al. (2021)
* H. limacinus*	AMB18196	Italy	KY549141	—	—	Papetti unpublished
* H. limosus*	MPU814001/ML81113HL2*	Cyprus	MT981620	—	—	Bellanger et al. (2021)
* H. lucorum*	MHKMU HHY-553	Jilin, China	MW762989	MW762777	OK063802	Huang et al. (2022a)
* H. lucorum*	HMAS 281284	Inner Mongolia, China	OK001727	OK001720	OK063803	Huang et al. (2022a)
* H. marzuolus*	—	Spain	KR817258	—	—	Pera & Mercado unpublished
* H. magnisporus*	HKAS 49094	Sichuan, China	PX868478	PX868376	PX907484	This study
* H. marcocontui*	KATO Fungi2976*	Turkey	MT981608	—	—	Bellanger et al. (2021)
* H. megasporus*	MICH34186	USA	MT981617	—	—	Bellanger et al. (2021)
* H. meridionalis*	LIP 0400300*	Cyprus	MG882081	—	—	Moreau et al. (2018)
* H. mesotephrus*	K(M)227410*	England	MT981695	—	—	Bellanger et al. (2021)
* H. monticola*	TENN:023684	USA	HQ185704	—	—	Matheny & Wolfenbarger unpublished
* H. murinidiscus*	GDGM82183*	Sichuan, China	OP547663	OP558656	OR575279	Wang et al. (2023)
* H. murinidiscus*	GDGM82186	Sichuan, China	OP547664	OP558657	OR575280	Wang et al. (2023)
* H. nemoreus*	LAS85112	Sweden	EF395374	—	—	Jacobsson and Larsson (2007)
* H. neoerubescens*	HMAS 253980	Sichuan, China	PX868479	PX868377	—	This study
* H. neoerubescens*	HMAS 254980	Tibet, China	PX868480	—	—	This study
* H. neoerubescens*	GDGM84466	Sichuan, China	OP547695	OP558687	OR575260	Wang et al. (2023)
* H. neoerubescens*	GDGM84603	China	OP547717	OP558709	OR575261	Wang et al. (2023)
* H. occidentalis*	MICH10917*	USA	MZ576439	—	—	Bellanger et al. (2021)
* H. ochraceodiscus*	MHKMU HT-489	Yunnan, China	PX868481	PX868378	PX907485	This study
* H. ochraceodiscus*	MHKMU HT-490*	Yunnan, China	PX868482	PX868379	PX907486	This study
* H. odoratus*	TENN 023658	USA	HQ185705	—	—	Matheny & Wolfenbarger unpublished
* H. orientalis*	HKAS 75586*	Hubei, China	MW290176	MW290239	MW928555	Huang et al. (2021a)
* H. orientalis*	CFSZ 20884	Inner Mongolia, China	MW290182	MW928625	MW928556	Huang et al. (2021a)
* H. olivaceoalbus*	GB0183666/EL196-10*	Sweden	MN243170	—	—	Bellanger et al. (2021)
* H. olivaceoalbus*	ectomycorrhiza S15	Germany	AF430253	AF430274	—	Haug (2002)
* H. olivaceoalbus*	KR6419	Germany	AF430252	AF430271	—	Haug (2002)
* H. orientimarzuolus*	LJW2439 (GDGM92361)	Yunnan, China	OP547754	OP558738	—	Wang et al. (2023)
* H. orientipurpurascens*	GDGM83073*	Yunnan, China	OP547678	OP558671	OR575288	Wang et al. (2023)
* H. pallidoagathosmus*	GDGM84681	Sichuan, China	OP547724	OP558716	OR575289	Wang et al. (2023)
* H. pallidoagathosmus*	GDGM84702*	Sichuan, China	OP547728	OP558720	OR575290	Wang et al. (2023)
* H. pallidoaurantiacus*	GDGM82435*	Yunnan, China	OP547673	OP558666	—	Wang et al. (2023)
* H. pallidoaurantiacus*	GDGM79190	China	OP547654	OP558646	OR575281	Wang et al. (2023)
* H. pallidoaurantiacus*	GDGM82410	Yunnan, China	OP547670	OP558663	OR575282	Wang et al. (2023)
* H. pallidoflavodiscus*	MHKMU TLP-1704	Yunnan, China	PX868483	PX868380	PX907487	This study
* H. pallidoflavodiscus*	MHKMU TLP-1745	Yunnan, China	PX868484	PX868381	PX907488	This study
* H. pallidoflavodiscus*	MHKMU YSD-106	Yunnan, China	PX868485	PX868382	PX907489	This study
* H. pallidoflavodiscus*	HKAS 69650	Yunnan, China	—	PX868383	PX907490	This study
* H. pallidoflavodiscus*	HKAS 57585	Yunnan, China	—	PX868384	PX907491	This study
* H. pallidoflavodiscus*	MHKMU MM-461	Yunnan, China	PX868486	PX868385	PX907492	This study
* H. pallidoflavodiscus*	MHKMU NX-158	Yunnan, China	PX868487	PX868386	—	This study
* H. pallidoflavodiscus*	MHKMU TLP-3291	Yunnan, China	PX868488	PX868387	PX907493	This study
* H. pallidoflavodiscus*	MHKMU HHY-938	Yunnan, China	—	PX868388	—	This study
* H. pallidofulvus*	GDGM84501*	Sichuan, China	OP547700	OP558692	OR575285	Wang et al. (2023)
* H. pallidofulvus*	GDGM84502	Sichuan, China	OP547701	OP558693	OR575286	Wang et al. (2023)
* H. paludosoides*	MICH10922*	USA	MT981616	—	—	Bellanger et al. (2021)
* H. parvirussula*	MHKMU HHY-911	Yunnan, China	MW290195	MW290254	MW928558	Huang et al. (2021a)
* H. parvirussula*	MHKMU TLP-3420	Yunnan, China	MW290196	MW290255	MW928559	Huang et al. (2021a)
* H. paulus*	MHKMU TLP-2546*	Yunnan, China	PX868489	PX868389	PX907494	This study
* H. penarioides*	SJ94067*	Sweden	EF395370	—	—	Jacobsson and Larsson (2007)
* H. penarioides*	AH51058	Spain	PX868490	PX868390	PX907495	This study
* H. penarius*	BP100883	Hungary	MK088117	—	—	Zajta et al. (2019)
* H. piceae*	WTU-F-073129	USA	MT955132	—	—	Trudell & Gordon unpublished
* H. piceae*	TU118045	Estonia	UDB015303	—	—	https://unite.ut.ee/
* H. pinophilus*	MHKMU HHY-878	Yunnan, China	MZ605854	MZ605806	MZ614647	Huang et al. (2022b)
* H. pinophilus*	MHKMU ZWH-494	Yunnan, China	MZ605859	MZ605811	MZ614650	Huang et al. (2022b)
* H. ponderatus*	JLF9211	USA	ON259580	—	—	Frank unpublished
* H. poetarum*	BP20818	Hungary	MK088103	—	—	Zajta et al. (2019)
* H. pseudodiscoideus*	HMAS277088*	Sichuan, China	OP547744	OP558727	—	Wang et al. (2023)
* H. pseudodiscoideus*	KUN-HKAS 112568	Tibet, China	MW762879	MW763004	MW773443	He and Yang (2021)
* H. pseudohypothejus*	MHKMU HT-477*	Yunnan, China	MW762991	MW762779	OK063805	Huang et al. (2022a)
* H. pseudopurpurascens*	HKAS 50697	Yunnan, China	PX868491	—	—	This study
* H. pseudopurpurascens*	HKAS 71175	Yunnan, China	PX868492	—	—	This study
* H. pseudopurpurascens*	MHKMU HHY-181	Yunnan, China	PX868493	PX868391	—	This study
* H. pseudopurpurascens*	MHKMU HHY-182	Yunnan, China	PX868494	PX868392	PX907496	This study
* H. pseudopurpurascens*	MHKMU HHY-183	Yunnan, China	PX868495	PX868393	—	This study
* H. pseudopurpurascens*	MHKMU HHY-184	Yunnan, China	PX868496	PX868394	PX907497	This study
* H. pseudopurpurascens*	MHKMU TLP-3368	Yunnan, China	PX868497	PX868395	PX907498	This study
* H. pseudopurpurascens*	MHKMU HHY-842-1	Yunnan, China	PX868498	PX868396	—	This study
* H. pseudopurpurascens*	MHKMU HHY-842-2	Yunnan, China	PX868499	PX868397	—	This study
* H. pseudopurpurascens*	MHKMU HHY-868-1	Yunnan, China	PX868500	PX868398	PX907499	This study
* H. pseudopurpurascens*	MHKMU HHY-868-2	Yunnan, China	PX868501	PX868399	PX907500	This study
* H. pseudopurpurascens*	MHKMU TLP-3348	Yunnan, China	PX868502	PX868400	PX907501	This study
* H. pseudopurpurascens*	MHKMU PYJ-356	Yunnan, China	PX868503	PX868401	PX907502	This study
* H. pseudopurpurascens*	MHKMU ZWH-473	Yunnan, China	PX868504	PX868402	—	This study
* H. pseudopurpurascens*	MHKMU HT-463	Yunnan, China	PX868505	PX868403	PX907503	This study
* H. pseudopurpurascens*	MHKMU MM-755	Yunnan, China	PX868506	PX868404	PX907504	This study
* H. pseudopurpurascens*	MHKMU MM-758	Yunnan, China	PX868507	PX868405	—	This study
* H. pseudopurpurascens*	MHKMU HT-680	Yunnan, China	—	PX868406	—	This study
* H. pudorinus*	E00905351	Sweden	PX868508	PX868407	—	This study
* H. pudorinus*	HKAS 54882	Yunnan, China	PX868509	PX868408	—	This study
* H. pudorinus*	GDGM44321	Sichuan, China	OP547626	OP558616	OR575297	Wang et al. (2023)
* H. pudorinus*	GDGM44328	Sichuan, China	OP547630	OP558620	OR575299	Wang et al. (2023)
* H. pustulatoides*	DAOM984764/HRL2832*	Canada	MT981658	—	—	Bellanger et al. (2021)
* H. pustulatus*	GB0183665!/EL185-14*	Sweden	MN243180	—	—	Bellanger et al. (2021)
* H. purpurascens*	EMB_787/92	Italy	MF399424	—	—	Peintner et al. unpublished
* H. purpurascens*	SJ01003	Sweden	UDB000576	—	—	https://unite.ut.ee/
* H. qinggangjun*	MHKMU YSD-20	Yunnan, China	MW290149	MW290220	MW928545	Huang et al. (2021a)
* H. qinggangjun*	MHKMU MM-464	Yunnan, China	MW290155	MW290222	MW928546	Huang et al. (2021a)
* H. queletii*	HMAS 290046	Inner Mongolia, China	MZ605862	MZ605813	MZ614652	This study
* H. roseobrunneus*	src414	USA	EF559268	—	—	Smith et al. (2007)
* H. roseodiscoideus*	PAM00103101*	France	MZ576440	—	—	Bellanger et al. (2021)
* H. roseoviolaceus*	SAAS4509*	Chongqing, China	OQ860087	OQ867285	—	Wang et al. (2023)
* H. russula*	AH 37145	Spain	MW290206	MW290261	—	Huang et al. (2021a)
* H. russula*	IB19630793	Switzerland	MF399427	—	—	Peintner et al. unpublished
* H. russula*	iNaturalist 31828832	USA	MN498103	—	—	Clements & Tighe unpublished
* H. russula*	4433	Canada	KM248883	—	—	Berube et al. unpublished
* H. russula*	GO-2009-116	Mexico	KT875017	—	—	Garibay et al. unpublished
* H. russula*	CLO-4280	Belize	KF381523	—	—	Lodge et al. (2014)
* H. russuliformis*	MICH 10939	USA	MF399455	—	—	Peintner et al. unpublished
* H. rutilans*	GDGM89538*	Yunnan, China	OQ860086	OQ867284	—	Wang et al. (2023)
* H. scabrellus*	LAH 35245*	Pakistan	MK066234	MK066238	—	Naseer et al. (2019)
* H. shennongjiaensis*	HKAS 75686	Hubei, China	PX868510	PX868409	—	This study
* H. shennongjiaensis*	HKAS 75704*	Hubei, China	PX868511	PX868410	PX907505	This study
* H. shennongjiaensis*	HKAS 75561	Hubei, China	PX868512	PX868411	PX907506	This study
* H. siccipes*	TENN23665	USA	MH087015	—	—	Larsson unpublished
* H. sichuanensis*	GDGM84471*	Sichuan, China	OP547699	OP558691	OR575305	Wang et al. (2023)
* H. sichuanensis*	GDGM84546	Sichuan, China	OP547709	OP558701	OR575306	Wang et al. (2023)
* H. sordidus*	GDGM70065	USA	OP547644	OP558635	OR575308	Wang et al. (2023)
* H. sordidus*	FLAS-F-60227	USA	MF153001	—	—	Kaminsky et al. unpublished
* H. sordidus*	AHSmith91580	USA	EF395373	—	—	Jacobsson and Larsson (2007)
* Hygrophorus * sp.	ECM114	Zhejiang, China	JQ991739	—	—	Qiong & Guo unpublished
* Hygrophorus * sp.	F32586	Canada	MH718228	—	—	Berbee et al. unpublished
* Hygrophorus * sp.	FLAS-F-60123	USA	MF153007	—	—	Kaminsky et al. unpublished
* Hygrophorus * sp.	FLAS-F-69167	USA	OP896813	—	—	Smith et al. unpublished
* Hygrophorus * sp.	GDGM70051	USA	OP547640	OP558631	OR575309	Wang et al. (2023)
* Hygrophorus * sp.	GDGM79241	Jilin, China	OP547659	OP558652	OR575310	Wang et al. (2023)
* Hygrophorus * sp.	GDGM82406	China	OP547669	OP558662	—	Wang et al. (2023)
* Hygrophorus * sp.	GDGM83568	Gansu, China	OP547687	OP558679	OR575311	Wang et al. (2023)
* Hygrophorus * sp.	GDGM84469	China	OP547697	OP558689	—	Wang et al. (2023)
* Hygrophorus * sp.	GL-2017	China	LT716040	—	—	Zhao et al. unpublished
* Hygrophorus * sp.	HKAS 56068	Yunnan, China	PZ413740	—	—	This study
* Hygrophorus * sp.	HKAS114478	China	MZ425996	—	—	Ge unpublished
* Hygrophorus * sp.	HMAS 280470	Tibet, China	PX868513	PX868412	PX907507	This study
* Hygrophorus * sp.	HRL1647	Canada	MT981622	—	—	Bellanger et al. (2021)
* Hygrophorus * sp.	IB19680126	Austria	MF399429	—	—	Peintner et al. unpublished
* Hygrophorus * sp.	IB19790638	Slovenia	MF399436	—	—	Peintner et al. unpublished
* Hygrophorus * sp.	inaturalist.org/observations/105475411	USA	OM349136	—	—	Singer unpublished
* Hygrophorus * sp.	iNAT:103385396	USA	OM403096	—	—	Jakob unpublished
* Hygrophorus * sp.	JR44	USA	KC791061	—	—	Taniguchi et al. (2018)
* Hygrophorus * sp.	Mushroom Observer 285692	USA	MG783389	MG493163	—	Clements unpublished
* Hygrophorus * sp.	Mushroom Observer 366634	USA	MN833651	—	—	Clements unpublished
* Hygrophorus * sp.	PAM19112303	Spain	MW172824	—	—	Bellanger et al. (2021)
* Hygrophorus * sp.	SAT-16-242-03	USA	MZ054342	—	—	Trudell et al. unpublished
* Hygrophorus * sp.	S.D. Russell iNaturalist 98567518	USA	OM987307	—	—	Russell unpublished
* Hygrophorus * sp.	TU110621	Papua New Guinea	UDB013227	—	—	https://unite.ut.ee/
* Hygrophorus * sp.	UBCF15697	Canada	FJ943242	—	—	Berbee & Kim unpublished
* Hygrophorus * sp.	XML1910693	China	OP547780	OP558768	—	Wang et al. (2023)
* Hygrophorus * sp.	YJ27	China	OM867700	—	—	Chen unpublished
* H. speciosus*	MHKMU HHY-1010	Jilin, China	MW762993	MW762781	OK063806	Huang et al. (2022a)
* H. suaveolens*	GB-0159606!*	Sweden	MH939180	—	—	Larsson et al. (2018)
* H. subcapreolarius*	GDGM45123	Sichuan, China	OP547636	OP558626	OR575312	Wang et al. (2023)
* H. subcapreolarius*	GDGM83194*	Yunnan, China	OP547684	OP558676	OR575314	Wang et al. (2023)
* H. subsalmonius*	4756-HRL 1323	Canada	KM248870	—	—	Berube et al. unpublished
* H. subviscifer*	SJ820927	Sweden	UDB000578	—	—	https://unite.ut.ee/
* H. tennesseensis*	TENN010922*	USA	NR119685	—	—	Schoch et al. (2014)
* H. unicolor*	SJ97046	Sweden	AY242857	—	—	Larsson and Jacobsson (2004)
* H. vividus*	GDGM82385	Yunnan, China	OP547667	OP558660	OR575315	Wang et al. (2023)
* H. whitei*	TENN024107	USA	MN243183	—	—	Bellanger et al. (2021)
* H. xiangjun*	MHKMU PYJ-407	Yunnan, China	MW762978	MW762767	OK063795	Huang et al. (2022a)
* H. xiangjun*	MHKMU HHY-817*	Yunnan, China	MW762979	MW762768	OK063796	Huang et al. (2022a)
* H. yunnanensis*	MHKMU HHY-322*	Yunnan, China	MW290214	MW290273	MW928564	Huang et al. (2021a)
* H. yunnanensis*	MHKMU TLP-2772	Yunnan, China	MW290211	MW290268	MW928561	Huang et al. (2021a)
* H. yadigarii*	KATO:Fungi:3843*	Turkey	MF370227	MF370228	—	Sesli et al. (2018)
* H. yukishiro*	TUMH:61715*	Japan	LC270641	—	—	Endo et al. (2018)
* H. yukishiro*	SAAS4870	Sichuan, China	OQ860088	OQ867286	—	Wang et al. (2023)
* H. yukishiro*	MHKMU HHY-1011	Henan, China	PX868514	PX868413	—	This study
* H. yukishiro*	MHKMU HHY-1012	Henan, China	PX868515	PX868414	PX907508	This study
* H. yukishiro*	MHKMU HHY-1172-1	Zhejiang, China	PX868516	PX868415	PX907509	This study
* H. yukishiro*	MHKMU HHY-1172-2	Zhejiang, China	PX868517	PX868416	PX907510	This study
* H. yukishiro*	MHKMU MJ-44	Anhui, China	PX868518	PX868417	—	This study

ML analysis was performed in RAxML v.7.0.3 (Stamatakis et al. 2008) under the GTRGAMMA model of nucleotide evolution, with branch supported through 1000 rapid bootstrap replicates. BI was conducted in MrBayes v.3.2 (Ronquist et al. 2012) via the CIPRES Science Gateway (Miller et al. 2010). For BI, the concatenated dataset was partitioned into three gene regions: ITS (positions 1–813), nrLSU (positions 814–1785) and *tef1-α* (positions 1786–2387). The best-fit substitution models for each partition were selected using PartitionFinder 2 (Lanfear et al. 2016) under the Akaike Information Criterion, resulting in GTR+I+G for ITS and nrLSU and SYM+I+G for *tef1-α*. Two independent runs of four simultaneous Markov chains each were performed for 20,000,000 generations for the ITS dataset and 9,000,000 generations for the concatenated dataset, sampling trees every 1000 generations. The average standard deviation of split frequencies fell below 0.01 upon completion. The initial 25% of sampled trees were discarded as burn-in after confirming stationarity using trace plots in Tracer v.1.7 (Rambaut et al. 2018).

**Figure 1.**
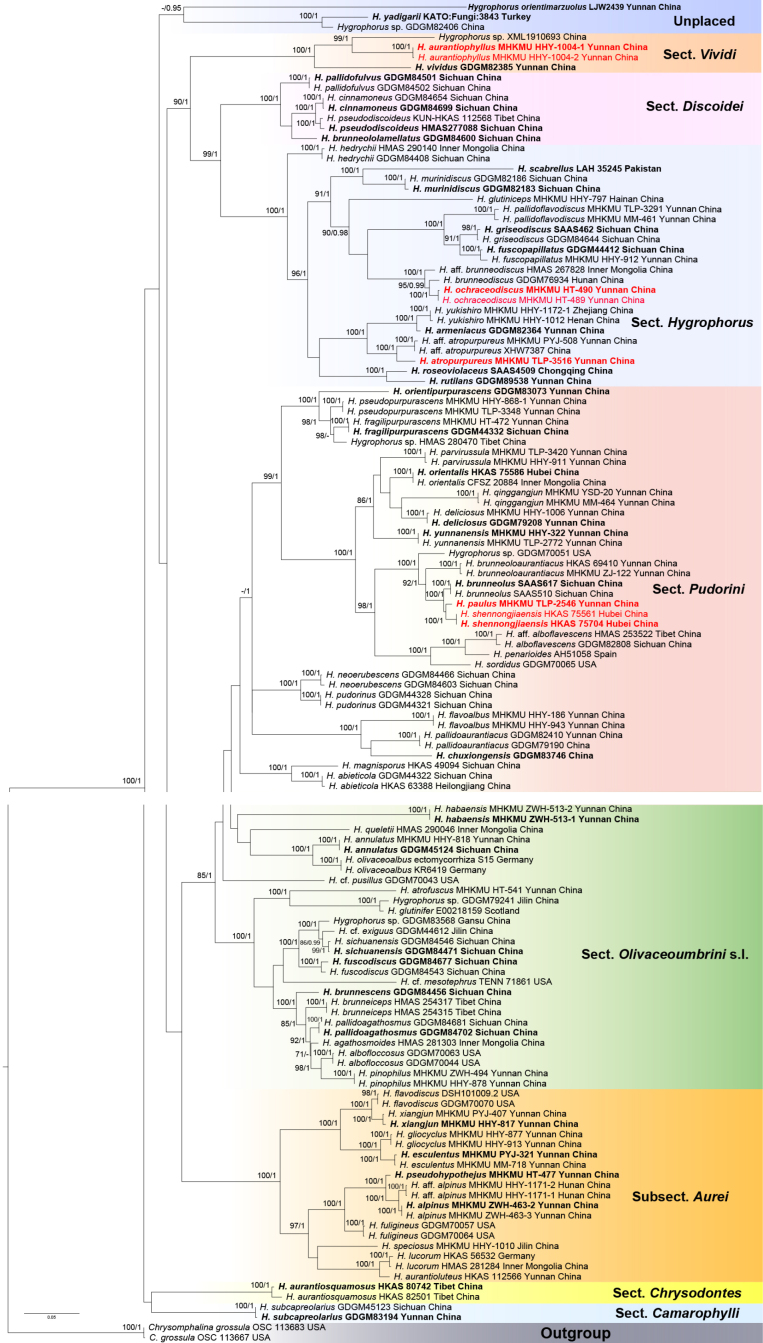
Phylogenetic tree of *Hygrophorus*, based on the concatenated dataset of ITS, nrLSU and *tef1-α*. RAxML BP values (≥ 70%) and Bayesian posterior probabilities (≥ 0.95) are shown above branches. New species are shown in red and type specimens are in bold.

Resulting trees were visualised in FigTree v.1.4.3 (Rambaut 2012) and graphically prepared using Adobe Illustrator (Fig. 1, Suppl. material 1). Clades with Bayesian posterior probabilities (BPP) ≥ 0.95 and ML bootstrap values (BS) ≥ 70 were considered statistically supported.

## Results

### Phylogenetic analyses

This study newly generated a total of 261 sequences, including 103 ITS, 97 nrLSU and 61 *tef1-α*. Additional sequences were retrieved from GenBank and UNITE. All newly-generated sequences are listed in Table 1. Two phylogenetic datasets were constructed: an ITS dataset comprising 195 sequences, in which *Chrysomphalina grossula* (Pers.) Norvell, Redhead & Ammirati and *C. aurantiaca* (Peck) Redhead were designated as outgroup taxa following Lodge et al. (2014); and a multilocus dataset including 131 ITS, 131 nrLSU and 110 *tef1-α* sequences, representing 84 taxa of *Hygrophorus*, with *C. grossula* as the outgroup. The final alignment consisted of 1086 characters for the ITS dataset and 2387 characters for the concatenated dataset (ITS: 813; nrLSU: 972; *tef1-α*: 602). The alignment is available at TreeBASE (Accession 32564).

Both Maximum Likelihood (ML) and Bayesian Inference (BI) analyses produced trees with largely congruent topologies for the ITS and concatenated datasets (Fig. 1, Suppl. material 1). The genus *Hygrophorus* was strongly supported as monophyletic in all analyses. Due to the absence of *rpb2* sequences for more than half of the included *Hygrophorus* species, this locus was excluded from the multi-locus phylogenetic reconstruction. In the resulting phylogenies, sections *Pudorini* and *Olivaceoumbrini* were not recovered as highly supported monophyletic clades, consistent with previous studies (e.g. Bellanger et al. (2021)). Within our phylogenetic framework, the examined specimens resolved into species-level lineages belonging to three sections. Here, we describe five new species: *H. atropurpureus* and *H. ochraceodiscus* in sect. *Hygrophorus*, *H. paulus* and *H. shennongjiaensis* in sect. *Pudorini* and *H. aurantiophyllus* in sect. *Vividi*.

Based on separate BLAST analyses of ITS and nrLSU sequences conducted on 16 May 2026, the top five most similar taxa with significant alignments are presented in Table 2.

**Table 2.** Summary of NCBI BLAST results for five new species, based on ITS and nrLSU sequences (accessed on 16 May 2026).

Taxon	Gene Locus	Top Ten Similar Taxa with Significant Alignments	Maximum Record Descriptions					References
			Max Score	Total Score	Query Cover	E value	Identity	
* Hygrophorus atropurpureus*	ITS	* Hygrophorus * sp. voucher XHW7387	833	833	51%	0.0	96.27%	Wang et al. (2023)
		* H. arbustivus*						Osmundson et al. (2013)
		* H. yukishiro*						Endo et al. (2018)
	nrLSU	* Hygrophorus * sp. voucher XHW7387	1607	1607	98%	0.0	99.66%	Wang et al. (2023)
		H. cf. arbustivus strain G1146						Varga et al. (2019)
		* H. yukishiro* voucher SAAS4870						Wang et al. (2023)
		* H. armeniacus*						Wang et al. (2023)
* H. aurantiophyllus*	ITS	* Hygrophorus * sp. voucher XML1910693	649	649	52%	0.0	89.79%	Wang et al. (2023)
		* H. piceae* isolate SAT-13-274-04						Trudell and Gordon unpublished
		* Serratia surfactantfaciens* strain YD25						Su et al. (2016)
		Uncultured *Hygrophorus* clone M251						Lang and Polle unpublished
		Uncultured fungus clone 3329L3						Taylor et al. (2014)
	nrLSU	* Hygrophorus * sp. voucher XML1910693	1461	1461	100%	0.0	96.92%	Wang et al. (2023)
		* H. vividus*						Wang et al. (2023)
		* Hygrophorus * sp. ‘sp-CA02’						Roy et al. unpublished
		* H. hedrychii* voucher GDGM84408						Wang et al. (2023)
* H. ochraceodiscus*	ITS	* H. brunneodiscus*	950	950	98%	0.0	97.17%	Wang et al. (2023)
	nrLSU	* H. brunneodiscus*	1570	1570	100%	0.0	99.88%	Wang et al. (2023)
* H. paulus*	ITS	* Hygrophorus * sp. voucher HKAS114478	911	911	100%	0.0	100.00%	Ge unpublished
		* H. brunneolus*						Wang et al. (2023)
		* Hygrophorus * sp. voucher OMS-084						Shirakawa et al. (2022)
		* Hygrophorus * sp. voucher FLAS-F-72303						Caiafa et al. unpublished
		* Hygrophorus * sp. ‘GA01’ isolate S.D. Russell iNaturalist 98567518						Russell unpublished
	nrLSU	* H. brunneolus*	1539	1539	100%	0.0	99.30%	Wang et al. (2023)
		* Hygrophorus * sp. voucher GDGM70051						Wang et al. (2023)
		* Hygrophorus * sp. voucher FLAS-F-72303						Caiafa et al. unpublished
		* H. brunneoloaurantiacus*						Wang et al. (2023)
		* H. russula* voucher AH19677						Huang et al. (2021a)
* H. shennongjiaensis*	ITS	Uncultured *Hygrophorus* clone ECM115	804	804	93%	0.0	99.11%	Qiong and Guo unpublished
		* Hygrophorus * sp. voucher HKAS114478						Ge unpublished
		* H. nemoreus*						Castro (2018)
		* H. nemoreus* voucher A30						Ouali and Sbissi unpublished
		* H. nemoreus* voucher LAS85112						Jacobsson and Larsson (2007)
	nrLSU	* H. brunneolus*	872	872	100%	0.0	98.98%	Wang et al. (2023)
		* Hygrophorus * sp. voucher GDGM70051						Wang et al. (2023)
		* H. brunneoloaurantiacus*						Wang et al. (2023)
		* Hygrophorus * sp. voucher FLAS-F-72303						Caiafa et al. unpublished
		* H. orientalis*						Wang et al. (2023)

### Taxonomy

#### *Hygrophorus* sect. *Hygrophorus*

##### Hygrophorus atropurpureus

Taxon classificationFungiAgaricalesHygrophoraceae

L.P. Tang & H.Y. Huang sp. nov.

6CE43A96-5D2B-5D14-91E3-7910B74046BE

861804

Fig. 2

###### Chinese name.

紫黑蜡伞.

###### Diagnosis.

*Hygrophorus atropurpureus* is distinguished from the closest relative, *H. arbustivus* by its smaller basidiomata, a blackish-purple pileus, scattered to gregarious habit and occurrence in the subalpine belt (2000–2700 m elevation) of Yunnan Province, south-western China.

**Figure 2.**
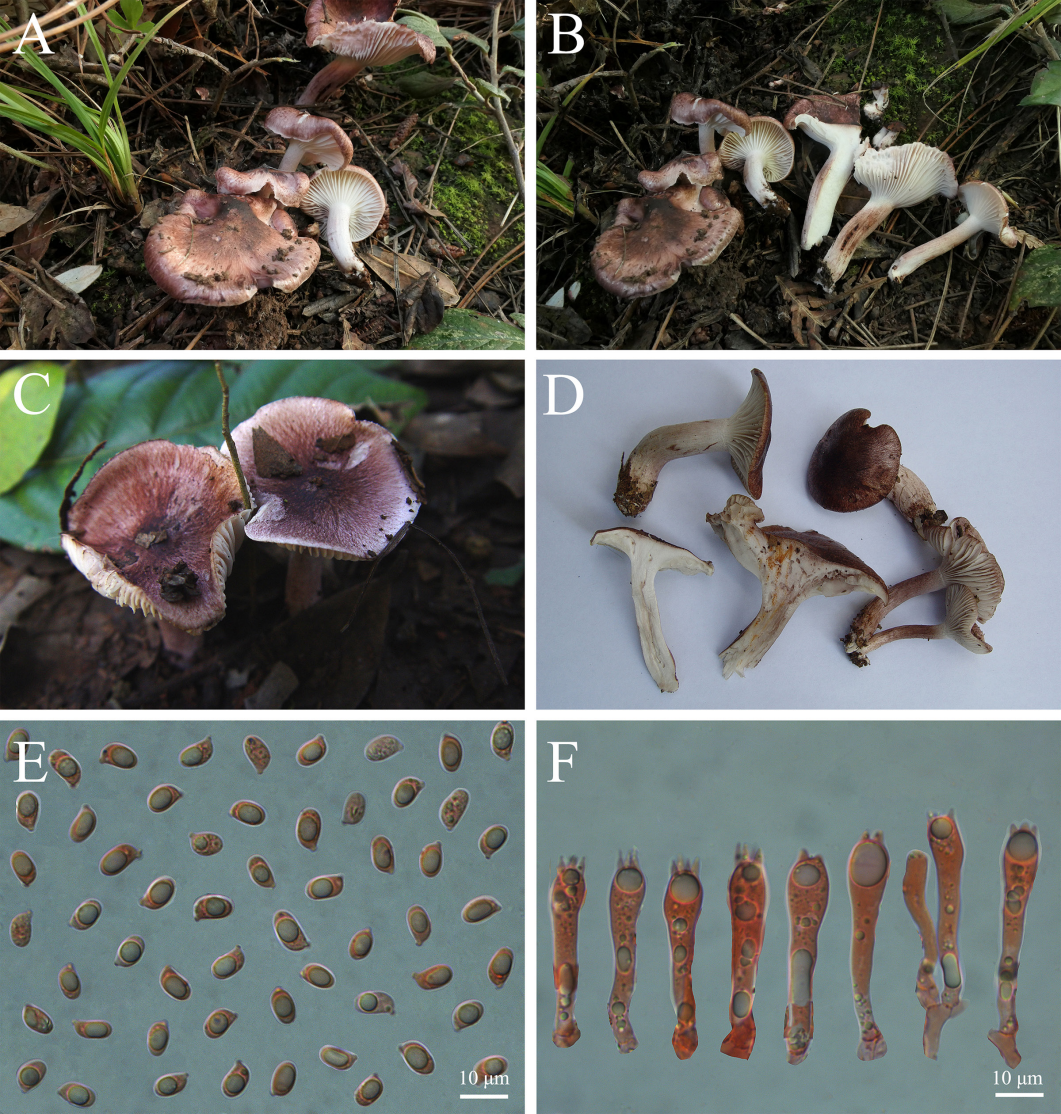
Macroscopic and microscopic features of *Hygrophorus atropurpureus*. **A–D**. Basidiomata (**A, B**. MHKMU TLP-3516, holotype; **C, D**. HKAS 49694); **E**. Basidiospores; **F**. Basidia.

###### Etymology.

The species epithet “*atropurpureus*” combines the Latin “*atro-*” (dark) and “*purpureus*” (purple), referring to the characteristic dark purple to purplish-black colouration of the pileus.

###### Type.

China • Yunnan Province: Eryuan County (洱源县), Ma’er Mountain (马耳山), in mixed broadleaf-conifer forests, dominated by *Quercus aquifolioides*, *Quercus* spp., *Pinus armandii*, *P. densata*, *Rhododendron decorum* and *Vaccinium fragile*, 26°5'25"N, 100°8'45"E, elev. 2700 m, 4 October 2020, *L.P. Tang 3516* (holotype: MHKMU TLP-3516, isotype: HMAS 354461; GenBank Acc. Nos.: ITS = PX868434, nrLSU = PX868336, *tef1-α* = PX907458).

###### Description.

***Basidiomata*** small to medium-sized, rather fragile. ***Pileus*** 2–4 cm diam., convex with an incurved margin when young, expanding to plane with a depressed centre; margin often wavy and uplifted with age. Colour purplish-black (12F4–5) when young, fading to purple, dark purple to blackish-purple (12D3–4) when mature, margin light purple (12B2–3). Surface dry, covered with tomentose squamules. Context white (1A1). ***Lamellae*** up to 0.3 cm wide, decurrent, subdistant, with 35–50 full lamellae per pileus; lamellulae numerous, narrowed; colour white (1A1). ***Stipe*** 3–4 cm long, 0.5–0.8 cm diam., central, cylindrical, equal; surface reddish-purple (12B2–3); context firm, white (1A1). ***Basal mycelium*** white. ***Odour*** not distinctive. ***Taste*** unrecorded.

***Basidiospores*** [60/3/2] 6.8–8.5 (–9) × 4–5 (–6) μm, Q = 1.45–1.86 (–2.13), Q_m_ = 1.66 ± 0.16, ellipsoid to oblong, smooth, thin-walled, hyaline, inamyloid, with a distinct hilar appendix. ***Basidia*** 30–50 × 7–10 μm, clavate, slender, thin-walled, 4-sterigmate; sterigmata up to 4 μm long. ***Hymenophoral trama*** slightly divergent, non-gelatinised, composed of cylindrical hyphae 3–15 μm diam. and inflated elements up to 25 μm diam., thin-walled, hyaline. ***Subhymenial layer*** next to hymenium, not gelatinised, composed of short cylindrical to irregularly-shaped elements 7–40 × 4–7 μm, thin-walled, hyaline. ***Pileipellis*** an ixotrichoderm, gelatinised, consisting of narrow, interwoven, thin-walled, hyphae 2–6 μm diam., branched or unbranched. ***Pileal trama*** of dense, thin-walled hyphae 6–18 μm diam. ***Stipitipellis*** a cutis, hyphae 3–5 μm diam., thin-walled. ***Stipititrama*** of parallel, thin-walled hyphae 3–21 μm diam. ***Mycelial hyphae*** 1.5–5 μm diam., smooth. ***Clamp connections*** present in all tissues.

###### Known distribution.

Yunnan Province, China.

###### Habit and habitat.

Scattered to gregarious in mixed broadleaf-conifer forests, dominated by *Pinus armandii*, *P. densata*, *Quercus aquifolioides*, *Quercus* spp., *Rhododendron decorum* and *Vaccinium fragile*, elev. 2000–2700 m; fruiting in autumn (September–October).

###### Additional material examined.

China • Yunnan Province: Kunming City (昆明市), Kunming Institute of Botany, Chinese Academy of Sciences, elev. 2000 m, 25 September 2005, *Z.W. Ge 929* (paratype: HKAS 49694).

###### Notes.

Phylogenetically, *H. atropurpureus* forms a well-supported monophyletic clade, together with an undescribed sister taxon labelled H. aff. atropurpureus from Yunnan Province and the European species *H. arbustivus* Fr. However, *H. arbustivus* is phylogenetically more distant to the two Chinese species and differs in its ochraceous-brown pileus and its occurrence in low-elevation European habitats (< 1500 m), where it is likely associated with *Quercus* (Candusso 1997; this study). Hygrophorus aff. atropurpureus is currently known from a single collection with suboptimal material; no obvious morphological differences from *H. atropurpureus* have been observed and a formal comparison will be deferred until additional specimens become available.

##### Hygrophorus fuscopapillatus

Taxon classificationFungiAgaricalesHygrophoraceae

C.Q. Wang & T.H. Li, Mycokeys 68: 58 (2020)

E06BAF60-1F4A-52D7-9B9F-73D5D3C3A133

570701

Fig. 3

###### Known distribution.

Sichuan and Yunnan Provinces, China.

###### Habit and habitat.

Scattered in broadleaf forests, occasionally forming arcs or fairy rings; likely associated with *Quercus guyavaefolia*; occurs at elevations of 2000–3500 m, fruiting from summer to autumn (July–September).

###### Material examined.

China • Yunnan Province: Eryuan County (洱源县), Ma’an Mountain Tianchi (马耳山天池), in broadleaf forests dominated by Ericaceae and *Quercus*, 26°15'22"N, 100°6'4"E, elev. 3500–3600 m, 22 August 2014, *L.P. Tang 1784* (MHKMU TLP-1784), *S.D. Yang* 87 (MHKMU YSD-87), *G.L. Zhang 112* (MHKMU ZGL-112); • Jianchuan County (剑川县), Shibao Mountain (石宝山), in broadleaf forests dominated by Ericaceae and Fagaceae, 26°23'49"N, 99°50'42"E, elev. 2500 m, 17 August 2014, *S.D. Yang 16* (MHKMU YSD-16); • *ibid*., 19 August 2014, *J. Zhao 29* (MHKMU ZJ-29); • *ibid*., 14 September 2019, *W.H. Zhang 206* (MHKMU ZWH-206); • Kunming City (昆明市), Shuanglong Town (双龙乡), Wild Duck Lake Park (野鸭湖公园), elev. 2000 m, 8 September 2008, *L.P. Tang 739* (HKAS 54976); • Lijiang City (丽江市), Jiuhe Town (九河乡), elev. 2780 m, 20 August 2010, *Wu 333* (HKAS 63564); • Lijiang Alpine Botanical Garden (丽江高山植物园), 26°58'26"N, 100°10'46"E, elev. 3185 m, 22 August 2020, *H.Y. Huang 856* (MHKMU HHY-856); • Shangri-La City (香格里拉市), Sanba Town (三坝乡), in mixed broadleaf-conifer forests dominated by Ericaceae and Fagaceae, under *Quercus guyavaefolia*, 27°23'12"N, 100°6'31"E, elev. 3100–3200 m, 25 August 2020, *H.Y. Huang 891* (MHKMU HHY-891), *T. Huang 496* (MHKMU HT-496), *Y.J. Pu 400* (MHKMU PYJ-400); • *ibid*., 26 August 2020, *H.Y. Huang 912* (MHKMU HHY-912), *M. Mu 795* (MHKMU MM-795), *L.P. Tang 3463* (MHKMU TLP-3463); • *ibid*., 29 July 2021, *Y.M. Gu 21* (MHKMU GYM-21), *T. Huang 637* (MHKMU HT-637), *W.H. Zhang 650* (MHKMU ZWH-650); • *ibid*., 30 July 2021, *H.Y. Huang 1050* (MHKMU HHY-1050), *T. Huang 647* (MHKMU HT-647), *L.P. Tang 3601* (MHKMU TLP-3601); • Shizong County (师宗县), Junzi Mountain (菌子山), in broadleaf forests dominated by Ericaceae, Juglandaceae and *Quercus*, 24°38'2"N, 104°8'59"E, elev. 2100–2400 m, 24 August 2018, *H.Y. Huang 161* (MHKMU HHY-161), *L.P. Tang 2579* (MHKMU TLP-2579), *L.P. Tang 2580* (MHKMU TLP-2580), *S.D. Yang 546* (MHKMU YSD-546); • *ibid*., 8 August 2019, *L.P. Tang 2694* (MHKMU TLP-2694); • *ibid*., 9 August 2019, *M. Mu 251* (MHKMU MM-251); • *ibid*., 11 August 2019, *H.Y. Huang 309* (MHKMU HHY-309), *M. Mu 279* (MHKMU MM-279); • *ibid*., 22 July 2020, *T. Huang 216* (MHKMU HT-216); • Wuding County (武定县), Gaoqiao Town (高桥镇), 25°38'53"N, 102°06'29"E, elev. 2520 m, 22 August 2016, *J. Wei 25* (MHKMU WJ-25); • Yulong County (玉龙县), Lijiang Observatory (丽江天文台), 26°42'2"N, 100°1'58"E, elev. 3210 m, 20 August 2020, *L.P. Tang 3298* (MHKMU TLP-3298); • *ibid*., 21 August 2020, *X. Na 157* (MHKMU NX-157).

**Figure 3.**
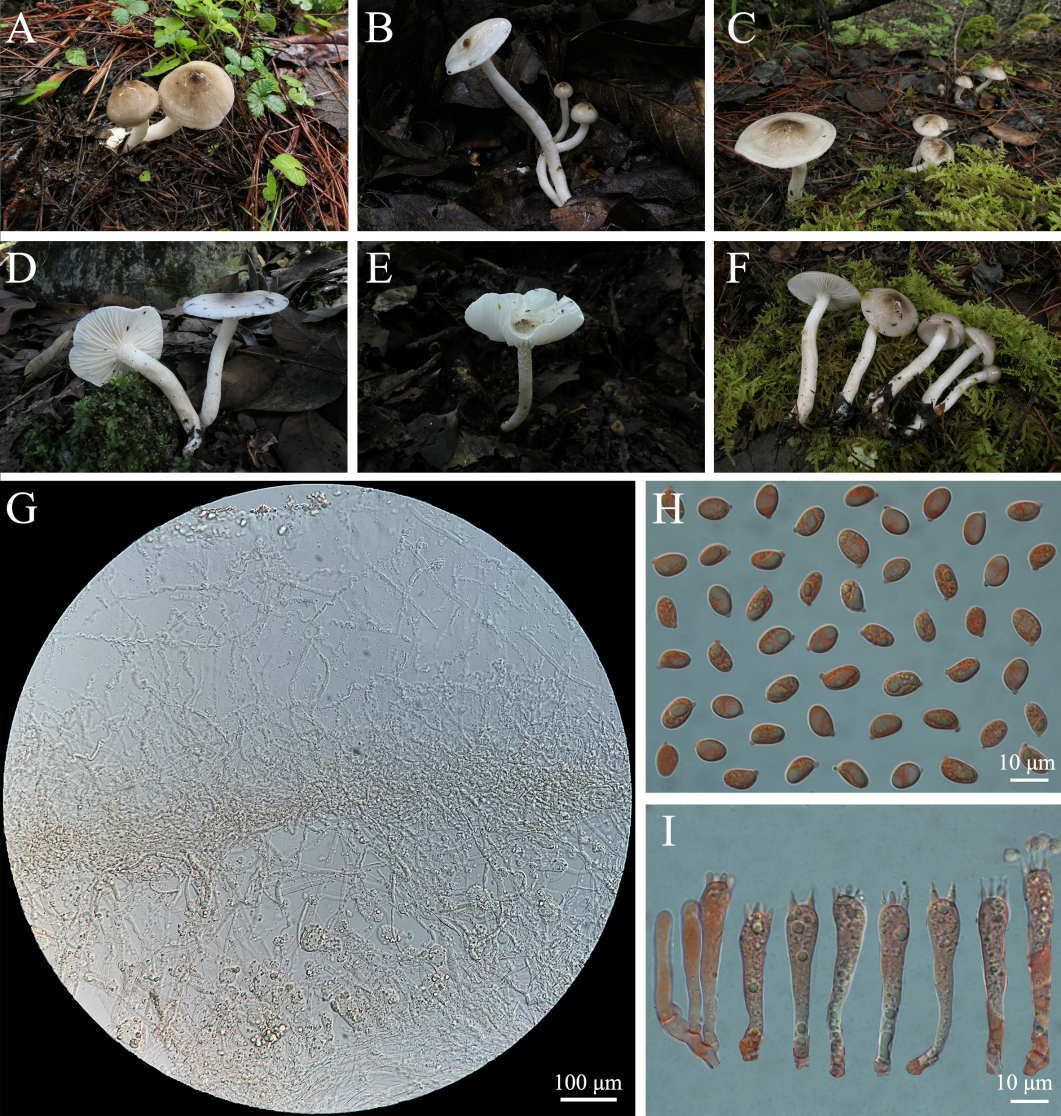
Macroscopic and microscopic features of *Hygrophorus fuscopapillatus*. **A–F**. Basidiomata (**A**. MHKMU ZWH-650; **B**. MHKMU HHY-161; **C, F**. MHKMU HHY-1050; **D**. MHKMU HT-216; **E**. MHKMU HHY-309); **G**. Pileipellis; **H**. Basidiospores; **I**. Basidia.

###### Notes.

*Hygrophorus fuscopapillatus*, originally described from Sichuan, is distinguished by a combination of macro- and micro-morphological features. Its basidiomata are slender, with a pileus 2–7 cm diam. and a stipe measuring 5.5–10 × 0.4–1 cm. The pileus exhibits a greyish-brown disc and the lamellae are distant (25–35 full lamellae per pileus). A distinctive odour reminiscent of *H. cossus* (similar to the scent of goat moth larvae, *Cossus cossus*) is also characteristic. Microscopically, it produces ellipsoid basidiospores measuring 8–10 × 5–6 μm and the pileipellis is an ixotrichoderm, composed of subglobose, cylindrical or clavate elements that are insoluble in 5% KOH. Ecologically, this species grows scattered in broadleaf forests, occasionally forming arcs or fairy rings and is likely associated with *Quercus guyavaefolia*. It is currently known only from the subalpine zone (2000–3500 m) of south-western China (Wang et al. 2020, 2023; this study).

##### Hygrophorus glutiniceps

Taxon classificationFungiAgaricalesHygrophoraceae

C.Q. Wang & T.H. Li, Mycokeys 68: 60 (2020)

11593E61-9F54-5384-8233-88B83F3AC3D7

570663

Fig. 4

###### Known distribution.

Guangdong, Hainan, Hunan and Jiangxi Provinces, China.

###### Habit and habitat.

Gregarious to scattered, sometimes forming arcs or fairy rings, in broadleaf forests dominated by *Castanopsis fissa*, *C. indica*, *Lasianthus japonicus* and other Fagaceae. Occurs at elevations below 700 m, fruiting from summer to autumn (June–September).

###### Material examined.

China • Hainan Province: Baisha County (白沙县), Yingge Ridge Conservation Area (鹦哥岭自然保护区), in broadleaf forests dominated by *Castanopsis indica*, Fagaceae and *Lasianthus japonicus*, 19°1'15"N, 109°25'42"E, elev. 580 m, 14 August 2020, *H.Y. Huang 797* (MHKMU HHY-797).

**Figure 4.**
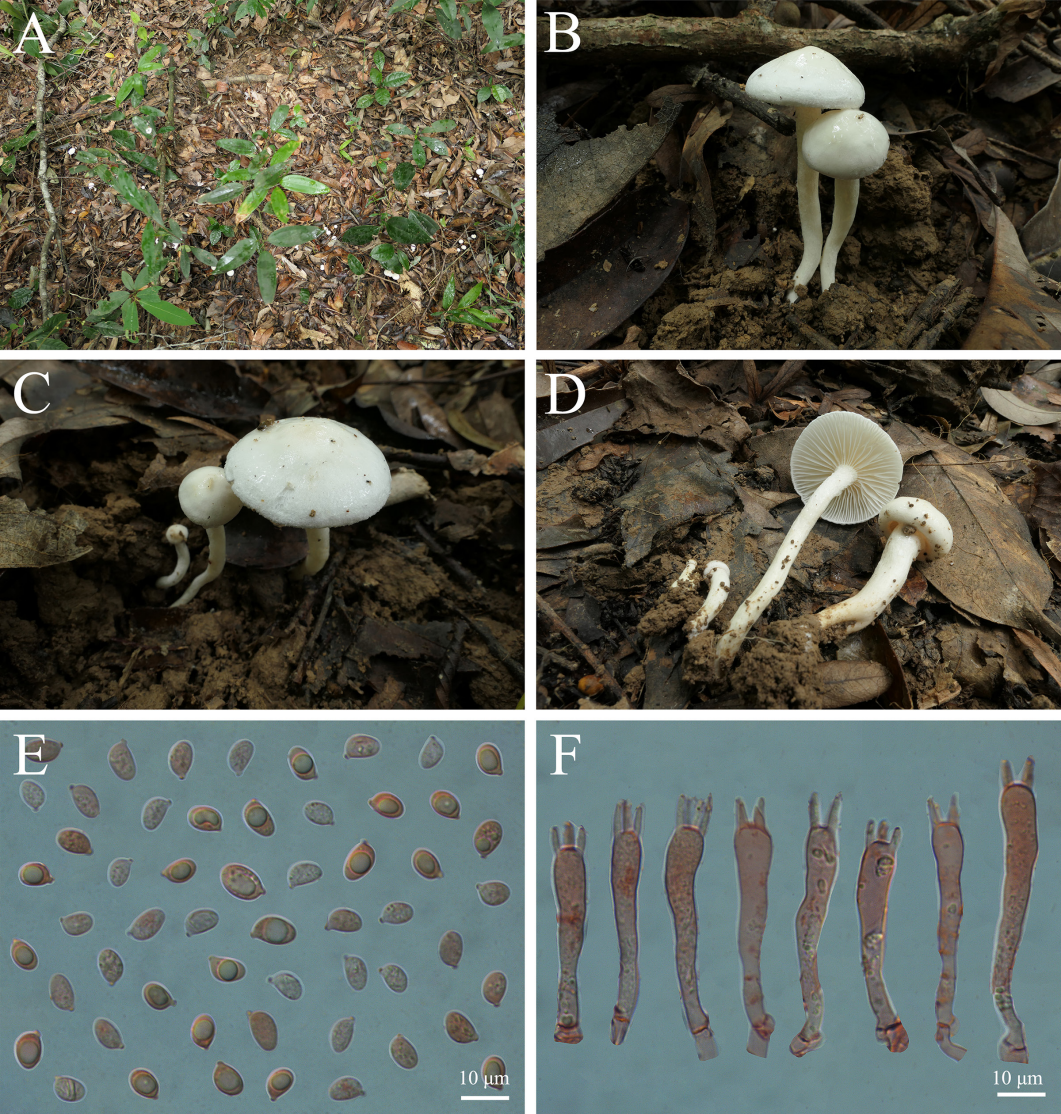
Macroscopic and microscopic features of *Hygrophorus glutiniceps* (MHKMU HHY-797). **A–D**. Basidiomata; **E**. Basidiospores; **F**. Basidia.

###### Notes.

*Hygrophorus glutiniceps*, originally described from Guangdong, is characterised by its white, sticky pileus and stipe and whitish lamellae that may turn ochraceous to brownish with age. It produces broadly ellipsoid to ellipsoid basidiospores measuring 7–9 × 4.5–5.5 µm and short basidia (31–47 × 6–9 μm). This species occurs scattered to gregarious, at times in arcs or fairy rings, at low elevations (< 700 m) in subtropical to tropical regions of China and is considered likely associated with *Castanopsis* (Wang et al. 2020, 2023; this study).

##### Hygrophorus hedrychii

Taxon classificationFungiAgaricalesHygrophoraceae

(Velen.) K. Kult, Česká Mykol. 10(4): 232 (1956)

93E2B345-70E6-54AA-B636-B6E1C55C28DD

298694

Fig. 5

###### Known distribution.

Heilongjiang, Inner Mongolia Autonomous Region, Shanxi and Sichuan Provinces, China; Europe.

###### Habit and habitat.

Scattered to gregarious in broadleaf forests dominated by *Betula*. In China, it occurs at elevations of 1000–3500 m; fruiting from late summer to autumn (August–September).

###### Material examined.

Sweden • Borgsjö sn., 30 August 1993, *R. Watling 25546* (E00905335). United Kingdom • England: North-West Yorkshire, 21 September 1986, *R. Watling 22946* (E00905313); • Scotland: South Aberdeenshire, elev. ca. 100 m, 3 September 1985, *R. Watling 18635* (E00905314). China • Heilongjiang Province: Suifenhe City (绥芬河市), Suifenhe National Forest Park (绥芬河国家森林公园), 26 September 2009, *T.Z. Wei 1135* (HMAS 250535); • Inner Mongolia Autonomous Region: Chifeng City (赤峰市), Ma’anshan National Forest Park (马鞍山国家森林公园), elev. unknown, 2 September 2019, *T.Z. Liu & Y.M. Gao* s. n. (CFSZ 21431); • Linxi County (林西县), Dalengshan Forest Farm (大冷杉林场), 44°5'22"N, 118°1'12"E, elev. 1300 m, 5 September 2018, *T.Z. Wei & Z.W. Peng & J.Y. Zhuang & T.Z. Liu 8883* (HMAS 281316), *T.Z. Wei & Z.W. Peng & J.Y. Zhuang & T.Z. Liu 8879* (HMAS 255531), *T.Z. Wei & Z.W. Peng & J.Y. Zhuang & T.Z. Liu 8866* (HMAS 290140).

**Figure 5.**
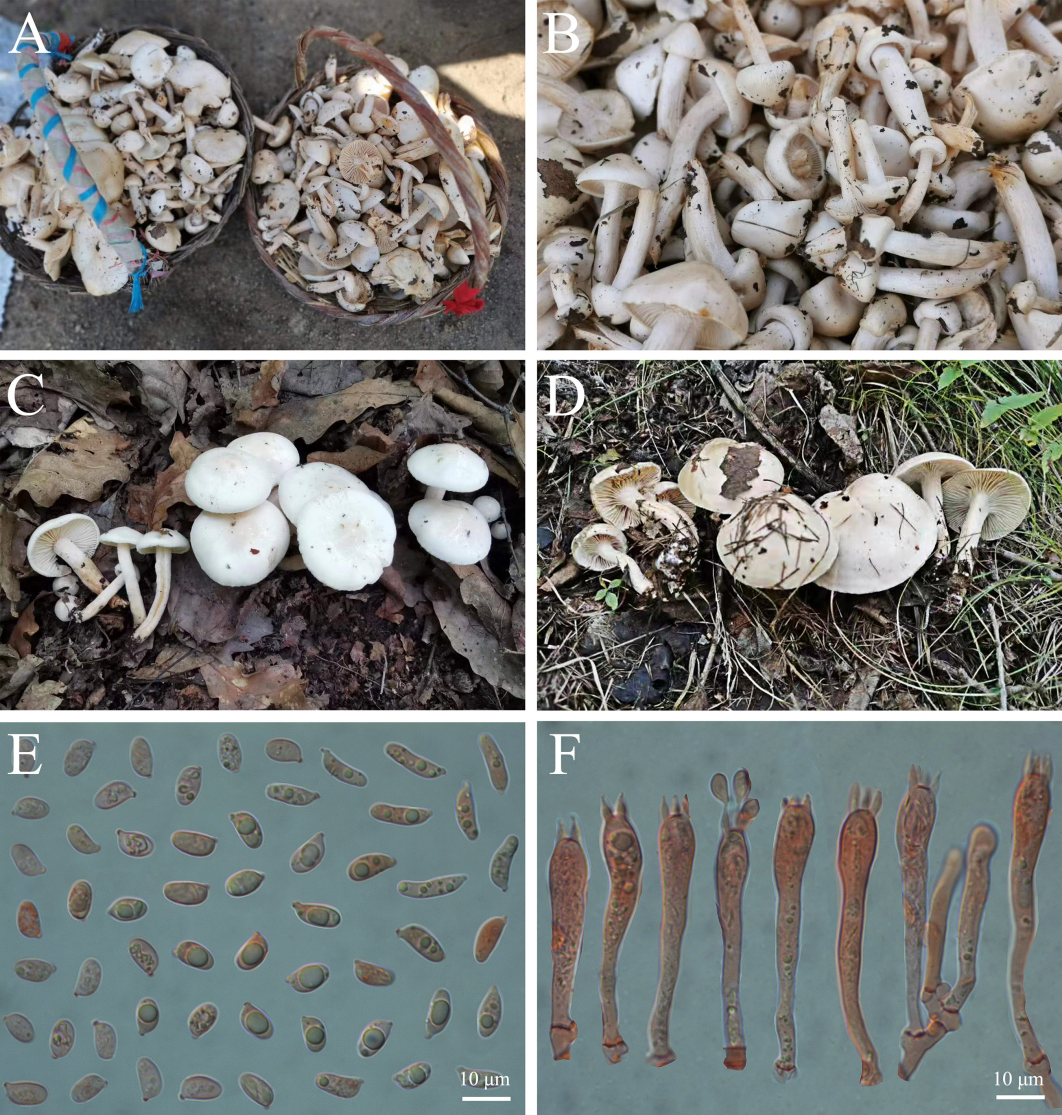
Macroscopic and microscopic features of *Hygrophorus hedrychii* (CFSZ 21431). **A–D**. Basidiomata; **E**. Basidiospores; **F**. Basidia.

###### Notes.

*Hygrophorus hedrychii*, originally described from Europe, is also widely distributed in China, where it is recognised as an edible mushroom in the Inner Mongolia Autonomous Region (Wang et al. 2020; this study). This species is distinguished by its white pileus and cream-coloured lamellae that develop pale ochraceous-pink tones at maturity. It produces ellipsoid to oblong, occasionally cylindrical basidiospores. Spore dimensions vary geographically: Chinese specimens measure 8–9 × 4–5 μm (Q = 1.6–2.0), European specimens 8–10 × 5–6 μm (Q = 1.49–1.96) and cylindrical forms 10–12.6 × 3–4 μm (Q = 2.5–4.25). Ecologically, it shows a wide elevational range (100–3500 m), occurring at low to middle elevations in Europe and north China (Heilongjiang, Inner Mongolia, Shanxi) and extends to high-altitude regions in southwest China (Sichuan) (Larsson and Jacobsson 2004; Campo 2015; Wang et al. 2020, 2023; this study).

##### Hygrophorus ochraceodiscus

Taxon classificationFungiAgaricalesHygrophoraceae

L.P. Tang & H.Y. Huang sp. nov.

B65D70C4-C35C-5F12-A9D6-569B0DED5BC3

861810

Fig. 6

###### Chinese name.

赭色蜡伞.

###### Diagnosis.

*Hygrophorus ochraceodiscus* differs from its closest relative, *H. brunneodiscus*, by its darker centre, short basidia (24–42 × 6–10 μm), solitary to scattered growth habit and distribution currently restricted to the subalpine zone (ca. 2800 m) of Yunnan Province, south-western China.

**Figure 6.**
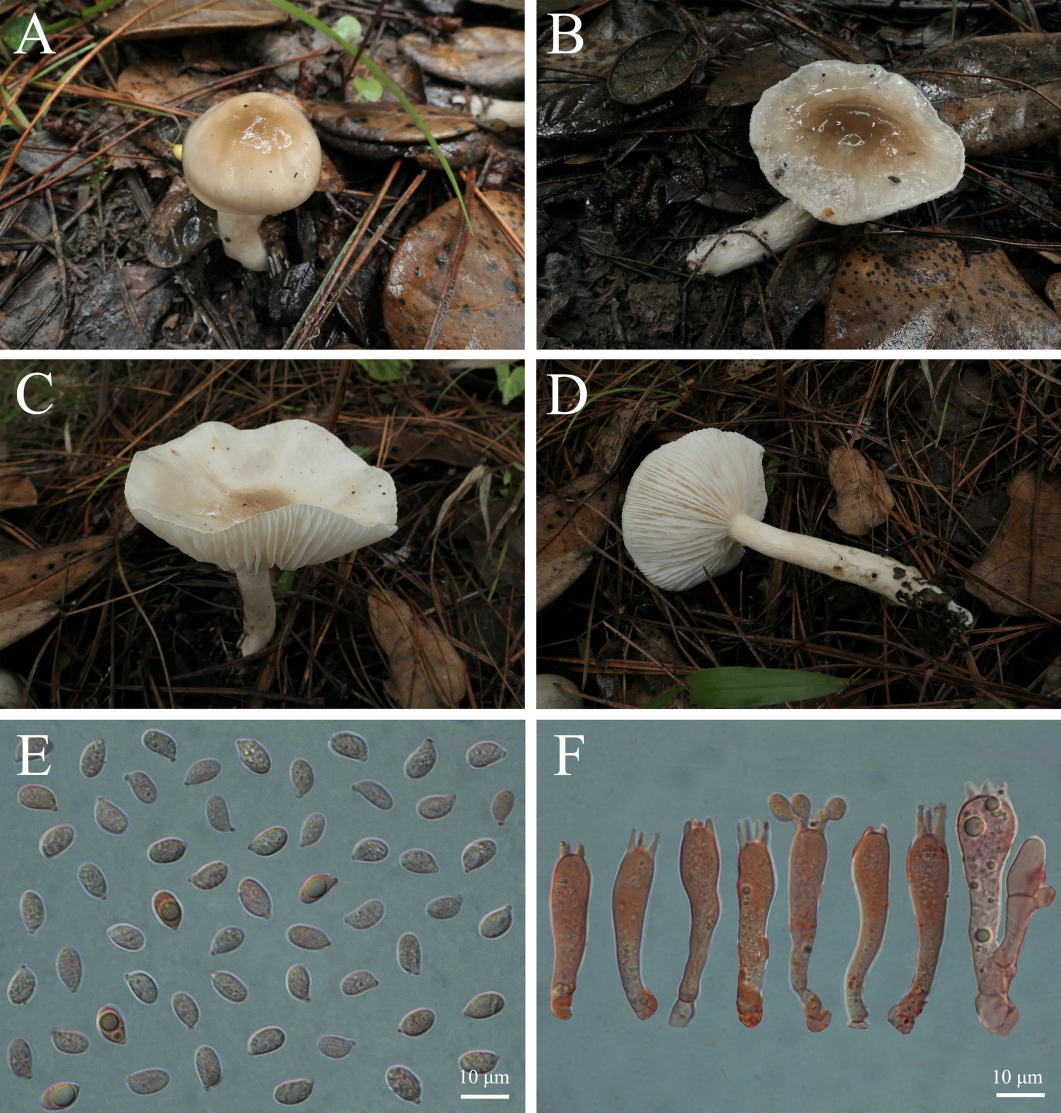
Macroscopic and microscopic features of *Hygrophorus ochraceodiscus*. **A–D**. Basidiomata (**A, B**. MHKMU HT-489; **C, D**. MHKMU HT-490, holotype); **E**. Basidiospores; **F**. Basidia.

###### Etymology.

Latin “*ochraceo-*” means ochraceous; “*discus*” means disc; “*ochraceodiscus*” refers to the characteristic ochraceous colouration of the pileus disc.

###### Type.

China • Yunnan Province: Shangri-La City (香格里拉市), Sanba Town (三坝乡), Mianshaba (绵沙坝), in mixed broadleaf-conifer forests dominated by Ericaceae, Fagaceae and *Pinus yunnanensis*, 27°21'14"N, 100°9'35"E, elev. 2755 m, 14 August 2020, *T. Huang 490* (holotype: MHKMU HT-490, isotype: HMAS 354462, GenBank Acc. Nos.: ITS = PX868482, nrLSU = PX868379, *tef1-α* = PX907486).

###### Description.

***Basidiomata*** medium-sized. ***Pileus*** 4.5–6 cm diam., nearly flat with a broad umbo; margin uplifted; pale brown (4B3–4), greyish-brown to yellowish-brown (4D4–6), margin brownish-white (4A2); surface viscid, covered with glutinous slime; context up to 0.3 cm thick, white (1A1). ***Lamellae*** 0.4–0.5 cm wide, adnate to decurrent, slightly close, with 50–60 full lamellae per pileus; lamellulae numerous, narrowed; white (1A1). ***Stipe*** 6–8 cm long, 0.8–0.9 cm diam., central, cylindrical, tapered towards base; apex covered with white punctations, overall sheathed by thick glutinous slime; surface white (1A1); context fibrous, white (1A1).

***Basidiospores*** [60/2/2] 7–9 (–9.5) × 4.5–5.5 (–6) μm, Q = 1.38–1.64 (–1.7), Q_m_ = 1.54 ± 0.08, ellipsoid, occasionally oblong, smooth, thin-walled, hyaline, inamyloid. ***Basidia*** 24–42 (–50) × 6–10 (–12) μm, clavate, slender, thin-walled, 4-sterigmate; sterigmata up to 5 μm long. ***Cystidioid elements on the lamellar edges and sides*** 24–45 × 3–8 μm, thin-walled, hyaline, scattered, apically pyriform, branched, flexuous, irregularly-shaped. ***Hymenophoral trama*** slightly divergent, non-gelatinised, composed of cylindrical hyphae (4–17 μm diam.) and inflated elements up to 28 μm diam., thin-walled, hyaline. ***Subhymenial layer*** next to hymenium, not gelatinised, composed of short cylindrical to irregularly-shaped elements, 6–39 × 4–9 μm, thin-walled, hyaline. ***Pileipellis*** an ixotrichoderm, gelatinised, consisting of narrow, interwoven hyphae 2–5 μm diam., thin-walled, branched or unbranched. ***Pileal trama*** of slightly parallel, dense hyphae 2–17 μm diam., thin-walled. ***Stipitipellis*** an ixotrichoderm, hyphae 2–8 μm diam., thin-walled. ***Floccules*** (at stipe apex) of compact, erect, branched hyphae 2.5–8 μm diam., with cylindrical to slender clavate terminal elements 25–70 × 4–7 μm, thin-walled. ***Stipititrama*** of parallel, thin-walled hyphae 3–20 μm diam. ***Mycelial hyphae*** 2–5 μm diam., smooth. ***Clamp connections*** present in all tissues.

###### Known distribution.

Yunnan Province, China.

###### Habit and habitat.

Solitary or scattered in mixed broadleaf-conifer forests dominated by Ericaceae, Fagaceae and *Pinus yunnanensis*; elev. ca. 2800 m; fruiting in late summer (August).

###### Additional material examined.

China • Yunnan Province: Shangri-La City (香格里拉市), Sanba Town (三坝乡), Mianshaba (绵沙坝), in mixed broadleaf-conifer forests dominated by Ericaceae, Fagaceae and *Pinus yunnanensis*, 27°21'14"N, 100°9'35"E, elev. 2755 m, 14 August 2020, *T. Huang 489* (paratype: MHKMU HT-489).

###### Notes.

This species is phylogenetically closely allied with *H. brunneodiscus* C.Q. Wang & T.H. Li, which it closely resembles morphologically. Although both species share similar basidiospores, *H. brunneodiscus* differs in having slightly smaller and more slender basidiomata (pileus 2–5 cm diam., stipe 4–9 × 0.4–0.7 cm), subdistant lamellae (36–40 full lamellae per pileus) and a distribution restricted to low-elevation habitats in Hunan and Shanxi Provinces (Wang et al. 2020, 2023; Zhang et al. 2024).

##### Hygrophorus pallidoflavodiscus

Taxon classificationFungiAgaricalesHygrophoraceae

C.Q. Wang, X.L. Gao & T.H. Li, Mycosphere 14: 1769 (2023)

C819AC80-5CD8-5D54-BBC0-A481FAD4BB1A

901128

Fig. 7

###### Known distribution.

Yunnan Province, China.

###### Habit and habitat.

Solitary or scattered in mixed broadleaf-conifer forests dominated by Ericaceae, Fagaceae, Theaceae and Pinaceae; likely associated with *Pinus densata* and *P. yunnanensis*; occurs at elevations of 2200–3300 m, fruiting during summer and autumn (July–September).

###### Material examined.

China • Yunnan Province: Eryuan County (洱源县), Ma’an Mountain (马鞍山), Huaneng Wind Farm (华能风电场), Shiputang station (石蒲塘站), elev. 3307 m, 24 August 2014, *S.D. Yang 106* (MHKMU YSD-106); • Jianchuan County (剑川县), Shibao Mountain (石宝山), in broadleaf forests dominated by Ericaceae and Fagaceae, 26°23'46"N, 99°50'53"E, elev. 2500–2600 m, 19 August 2014, *L.P. Tang 1704* (MHKMU TLP-1704); • *ibid*., 20 August 2014, *L.P. Tang 1745* (MHKMU TLP-1745); • *ibid*., 14 September 2019, *M. Mu 461* (MHKMU MM-461); • Lijiang City (丽江市), Gucheng District (古城区), Da Dong Town (大东乡), 18 August 2010, *Q. Zhao 929* (HKAS 69650); • Yulong County (玉龙县), Lijiang Observatory (丽江天文台), 26°42'2"N, 100°1'58"E, elev. 3210 m, 20 August 2020, *L.P. Tang 3291* (MHKMU TLP-3291); • *ibid*., 21 August 2020, *X. Na 158* (MHKMU NX-158); • Shangri-La City (香格里拉市), Sanba Town (三坝乡), Haba Snow Mountain (哈巴雪山), Mianshaba (绵沙坝), elev. 2800 m, 27 August 2020, *H.Y. Huang 938* (MHKMU HHY-938); • Yongping County (永平县), National Highway 320, elev. 2200 m, 30 July 2009, *Wu 54* (HKAS 57585).

**Figure 7.**
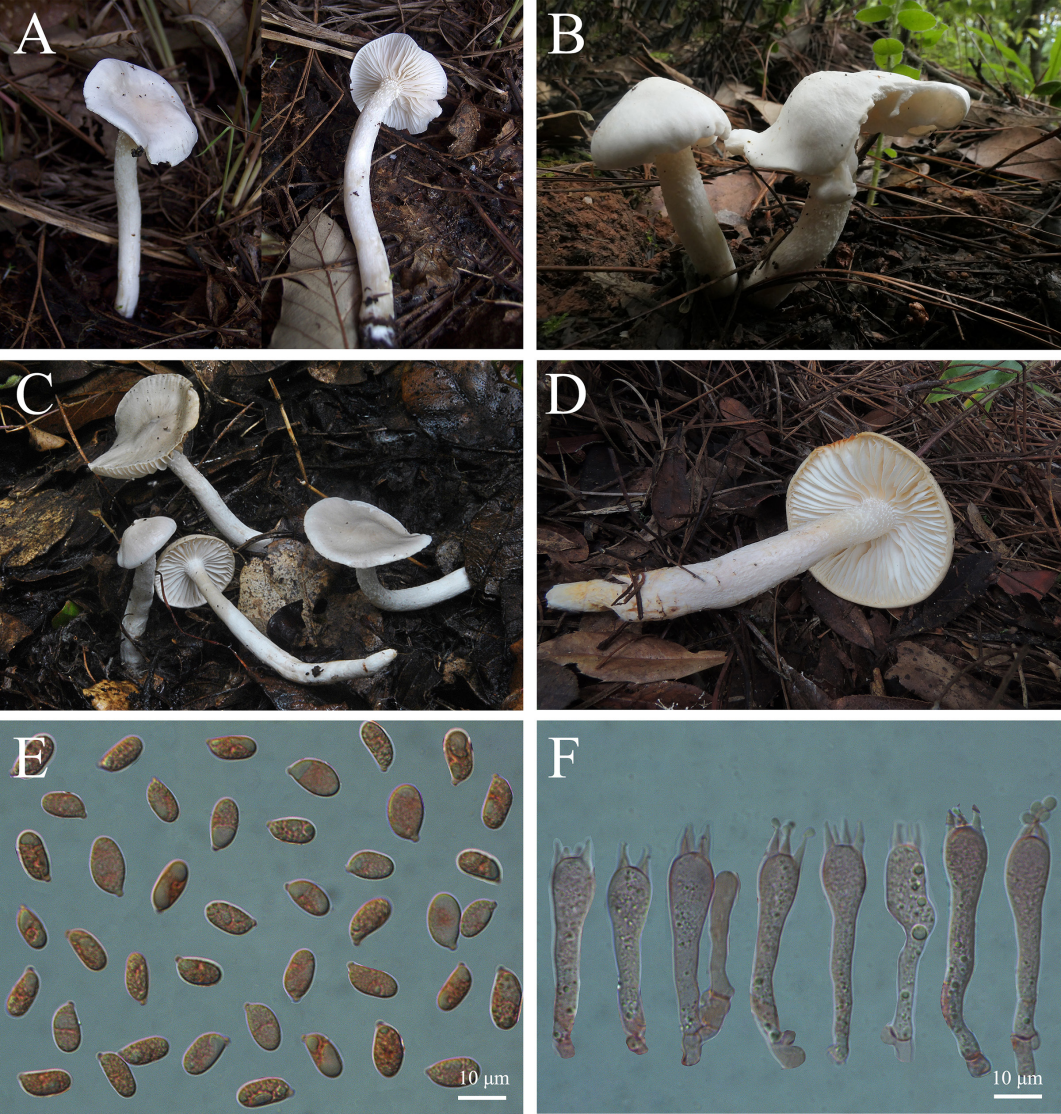
Macroscopic and microscopic features of *Hygrophorus pallidoflavodiscus*. **A–D**. Basidiomata (**A**. HKAS 57585; **B**. MHKMU MM-461; **C**. HKAS 69650; **D**. MHKMU YSD-106); **E**. Basidiospores; **F**. Basidia.

###### Notes.

*Hygrophorus pallidoflavodiscus* is distinguished by its slender basidiomata, with a pileus 2–6 cm diam. and a stipe measuring 6–9.5 × 0.5–1.3 cm. The pileus disc is pale grey to yellowish-white, and the context and lamellae may occasionally turn orangish when damaged. Additional diagnostic features include subdistant lamellae (35–50 full lamellae per pileus), ellipsoid to oblong basidiospores (9–11 × 5–6.2 μm, Q = 1.50–1.92), a solitary to scattered habit, a likely association with *Pinus densata* and *P. yunnanensis* and a distribution so far restricted to the subalpine belt (2200–3300 m) of Yunnan Province, south-western China (Wang et al. 2023; this study).

##### Hygrophorus yukishiro

Taxon classificationFungiAgaricalesHygrophoraceae

N. Endo, R. Tokoo & A. Yamada, in Endo, Tokoo, Fukuda & Yamada, Mycoscience 59(6): 450 (2018)

B51EA3DB-0DA8-5871-8560-5BA55983A389

821413

Fig. 8

###### Remark.

The following description is based primarily on Endo et al. (2018), with supplementary data on macro- and micromorphology, habit and distribution from our observations.

###### Description.

***Basidiomata*** small to medium-sized, fleshy. ***Pileus*** 2.5–6 cm diam., convex when young, expanding to broadly convex to plane with age; initially creamy to pale reddish-brown, maturing to brownish-red, darkening when dry, turning light pink after cooking, unchanged when damaged. ***Lamellae*** 0.3–0.7 cm wide, subdecurrent, slightly close, with 50–70 full lamellae per pileus; lamellulae numerous, narrowed; off-white to cream-white. ***Stipe*** 2.5–5 cm long, 0.8–2 cm diam., central, cylindrical, white; context firm, white. ***Odour*** and ***taste*** mild.

**Figure 8.**
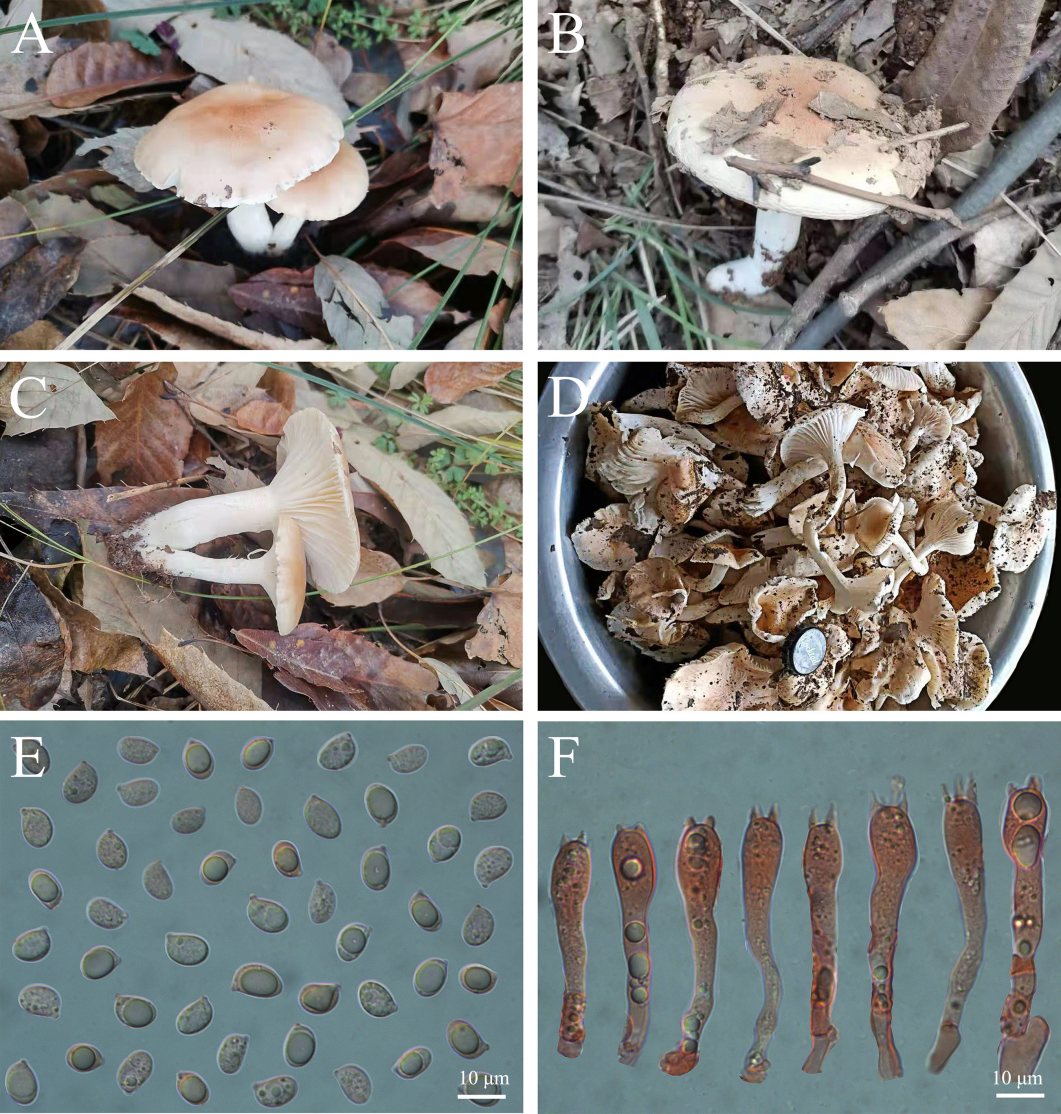
Macroscopic and microscopic features of *Hygrophorus yukishiro*. **A–D**. Basidiomata (**A–C**. MHKMU HHY-1012; **D**. MHKMU HHY-1011); **E**. Basidiospores; **F**. Basidia.

***Basidiospores*** [40/2/1] (7.5–) 8–10 × 5–6.5 (–7) μm, Q = 1.31–1.67 (–1.76), Q_m_ = 1.52 ± 0.11, ovoid to ellipsoid, occasionally oblong, smooth, thin-walled, hyaline, inamyloid. ***Basidia*** 38–53 (–64) × 7.5–10 μm, clavate, slender, thin-walled, 4-sterigmate; sterigmata up to 10 μm long. ***Hymenophoral trama*** slightly divergent, non-gelatinised, composed of cylindrical hyphae 3–20 μm diam. and inflated elements up to 32 μm diam., thin-walled, hyaline. ***Subhymenial layer*** next to hymenium, not gelatinised, composed of short elements, 12–40 × 3–7 μm, thin-walled, hyaline, cylindrical, irregularly-shaped. ***Pileipellis*** an ixotrichoderm, gelatinised, of narrow, closely interwoven hyphae 2–5 μm diam., thin-walled, branched or unbranched. ***Pileal trama*** of dense, thin-walled hyphae 4–30 μm diam. ***Stipitipellis*** a trichoderm, of hyphae 3–7 μm diam., thin-walled. ***Stipititrama*** of parallel, thin-walled, hyphae 4–17 μm diam. ***Mycelial hyphae*** 2–5 μm diam., smooth. ***Clamp connections*** present in all tissues.

###### Known distribution.

Anhui, Henan and Zhejiang Provinces, China; Japan.

###### Habit and habitat.

Gregarious in mixed broadleaf-conifer forests dominated by *Quercus*, likely associated with *Quercus serrata* and *Q. acutissima*; occurs at elevations below 1500 m, fruiting from late winter to spring (February–April).

###### Material examined.

China • Anhui Province: Chuzhou City (滁州市), elev. 300 m, 5 March 2022, *J. Ma 44* (MHKMU MJ-44). Henan Province: Xixia County (西峡县), elev. unknown, 14 March 2021, *H.Y. Huang 1011* (MHKMU HHY-1011); • Zhumadian City (驻马店市), elev. unknown, 25 March 2021, *H.Y. Huang 1012* (MHKMU HHY-1012). Zhejiang Province: Jinhua City (金华市), elev. unknown, 25 February 2022, *H.Y. Huang 1172* (MHKMU HHY-1172).

###### Notes.

*Hygrophorus yukishiro* was originally described from Japan and is widely distributed in China (Anhui, Henan and Zhejiang Provinces), where it is also recognised as an edible mushroom (Endo et al. 2018; this study). This species is characterised by a pale reddish-brown to brownish-red pileus, subdistant lamellae (50–70 full lamellae per pileus), ovoid to ellipsoid basidiospores measuring 8–10 × 5–6.5 μm (Q = 1.33–1.67) and a gregarious habit in *Quercus*-dominated mixed forests at low elevations (< 1500 m) during spring (February–April) (Endo et al. 2018; this study).

A Chinese collection from Sichuan Province (voucher SAAS4870) tentatively identified as *H. yukishiro* by Wang et al. (2023), exhibits notable morphological and ecological differences. These include a bright coloured (light yellow to orange) pileus, distant lamellae (32–44 full lamellae per pileus), smaller basidiospores (7–8 × 4.5–5 μm, Q = 1.4–1.6) and an autumn fruiting. Together with subtle ITS sequence variation, these features suggest that this specimen may represent a distinct, undescribed species. However, further research and additional collections from the region are necessary to confirm this taxonomic hypothesis.

#### *Hygrophorus* sect. *Olivaceoumbrini*

##### Hygrophorus queletii

Taxon classificationFungiAgaricalesHygrophoraceae

Bres., Fung. trident. 1: 11 (1881)

68A7BFE3-3B42-58DF-B8E7-CF340B9BFA5B

187237

Fig. 9

###### Known distribution.

Northern China (Inner Mongolia), Russia (GenBank Acc. No. MT302572) and Europe, where this species was originally described.

###### Habit and habitat.

Scattered to gregarious, occurring under *Larix decidua*; at elevations of 1000–1700 m; fruiting in autumn (September–October).

###### Material examined.

China • Inner Mongolia Autonomous Region, Chifeng City (赤峰市), Keshiketeng Banner (克什克腾旗), Huanggangliang National Forest Park (黄冈梁保护区), 43°36'39"N, 117°25'13"E, elev. 1490 m, 3 September 2018, *T.Z. Wei & Z.W. Peng & J.Y. Zhuang & T.Z. Liu 8705* (HMAS 290046).

###### Notes.

*Hygrophorus queletii* was originally described from European material (Bresadola 1881–1990; Candusso 1997) and has a broad Eurasian distribution (Candusso 1997; Campo 2015; Wang et al. 2023). The species is distinguished by its off-white basidiomata, often with faint to distinct pinkish tinges, oblong basidiospores measuring 9–10.5 (–11) × 5–6.8 μm, [Q = (1.44–) 1.54–2 (–2.1), Q_m_ = 1.82 ± 0.15], a stipitipellis with abundant gelatinous particles and the absence of clamp connections. It is likely associated with *Larix decidua* (Candusso 1997; Campo 2015; Wang et al. 2023; this study). The basidiospore dimensions of the Chinese specimen examined in this study align with those reported by Candusso (1997: 9–12.5 × 5–6.8 μm), but differ slightly from the measurements (7.0–10.0 × 4.0–5.5 μm) provided by Wang et al. (2023), possibly reflecting intraspecific variation. Notably, the absence of clamp connections — a feature shared with *H. adiaphorus* Hesler & A.H. Sm./*H. betulae* K. Bendiksen & E. Larss. and *H. glutinosus* Peck — is rare within the genus *Hygrophorus* (Hesler and Smith 1963; Candusso 1997; Larsson and Bendiksen 2020; Wang et al. 2023; this study).

**Figure 9.**
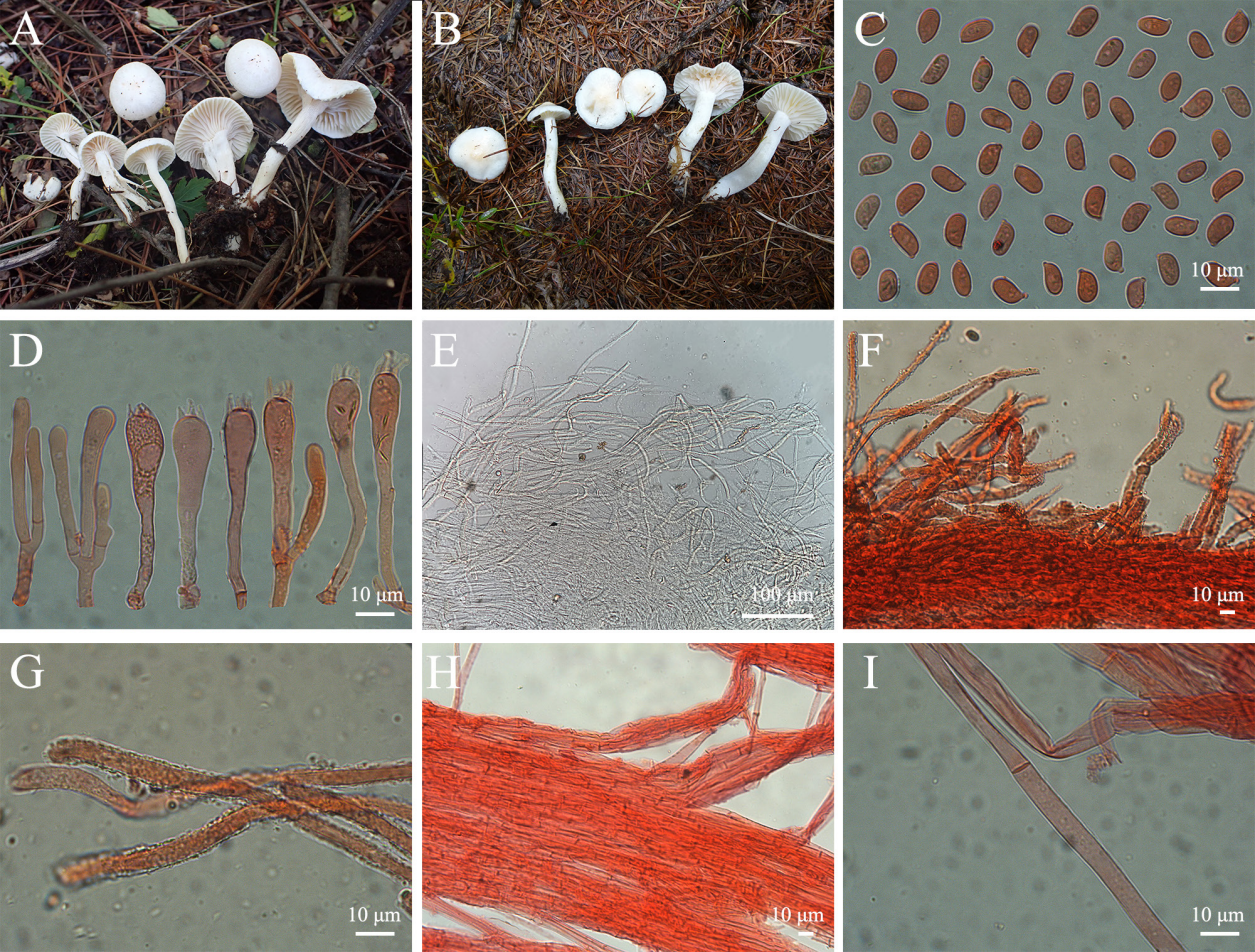
Macroscopic and microscopic features of *Hygrophorus queletii* (HMAS 290046). **A, B**. Basidiomata; **C**. Basidiospores; **D**. Basidia; **E**. Pileipellis; **F–G**. Floccule on stipe; **H, I**. Stipititrama.

#### *Hygrophorus* sect. *Pudorini*

##### Hygrophorus flavoalbus

Taxon classificationFungiAgaricalesHygrophoraceae

C.Q. Wang, X.H. Wang, L.Q. Mu & T.H. Li, Mycosphere 14: 1782 (2023)

D9CFCC80-0156-5BF2-8FB8-3304F62EF78E

MycoBank No: 901144

Fig. 10

###### Known distribution.

Guizhou and Yunnan Provinces, China.

###### Habit and habitat.

Scattered in forests dominated by *Lyonia ovalifolia* and *Pinus yunnanensis*; at elevations of 2100–2700 m; fruiting in autumn (September–October).

###### Material examined.

China • Guizhou Province: Guiyang City (贵阳市), elev. unknown, 17 October 2022, *J.G. Tian P299* (MHKMU TJG-P299). Yunnan Province: Heqing County (鹤庆县), Xiyi Town (西邑镇), in mixed broadleaf-conifer forest dominated by *Lyonia ovalifolia* and *Pinus yunnanensis*, 26°18'19"N, 100°11'2"E, elev. 2330 m, 2 October 2020, *H.Y. Huang 943* (MHKMU HHY-943), *H.Y. Huang 944* (MHKMU HHY-944); • Jianchuan County (剑川县), Laojunshan Town (老君山镇), 26°32'31"N, 99°35'13"E, elev. 2415 m, 3 October 2018, *H.Y. Huang 186* (MHKMU HHY-186).

###### Notes.

*Hygrophorus flavoalbus* is characterised by its robust basidiomata, with a pileus 3–12 cm diam. and a stipe measuring 3.5–8.5 × 1.3–3 cm. Additional distinguishing features include a yellowish to beige pileus, whitish dense lamellae and autumn fruiting under *Pinus yunnanensis* (Wang et al. 2023; this study). Originally described from Yunnan, its known distribution is here extended to Guizhou Province in south-western China. In both provinces, it is recorded as an edible mushroom.

**Figure 10.**
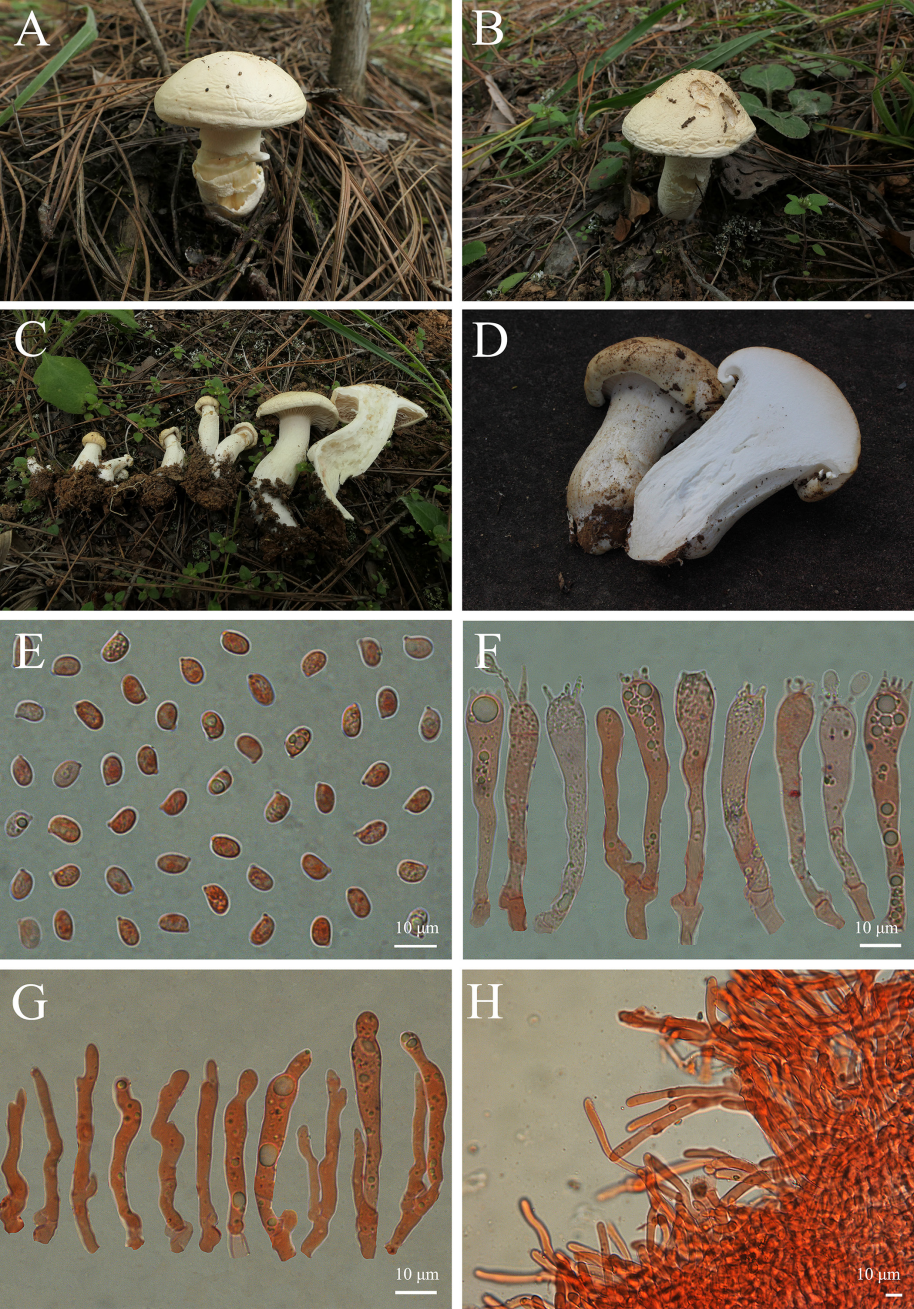
Macroscopic and microscopic features of *Hygrophorus flavoalbus*. **A–D**. Basidiomata (**A**. MHKMU HHY-944; **B, C**. MHKMU HHY-943; **D**. MHKMU HHY-186); **E**. Basidiospores; **F**. Basidia; **G**. Cystidioid elements on the lamellar; **H**. Floccule on stipe.

This study provides additional micromorphological details: cystidioid elements on the lamellae sides and edges measuring 20–52 × 3–7 μm, are thin-walled, hyaline, scattered and exhibit pyriform, flexuous, gourd-shaped, irregular or narrowly clavate apices, occasionally branched (Fig. 10G). Floccules on the stipe consist of compact, erect, branched hyphae with cylindrical to slender clavate terminal elements measuring 33–72 × 5–7 μm, thin-walled (Fig. 10H).

#### *Hygrophorus nemoreus* complex

**Notes**. The *Hygrophorus nemoreus* complex comprises at least eight phylogenetic species, including the Chinese taxa *H. brunneoloaurantiacus* C.Q. Wang, Ming Zhang & T.H. Li, *H. brunneolus* C.Q. Wang, Xiao Lan He & T.H. Li, *H. paulus* and *H. shennongjiaensis*; the European *H. nemoreus* (Pers.) Fr.; and three American sequences representing undescribed taxa. These taxa show distinct elevational zonation: *H. nemoreus* occurs scattered to gregarious at low elevations (< 1100 m) in Europe (Candusso 1997); *H. shennongjiaensis* is restricted to subtropical areas of central and eastern China below 2000 m; *H. paulus* is currently known only from Yunnan Province at ca. 2300 m (this study); *H. brunneolus* is endemic to high-elevation regions of Sichuan Province (ca. 3200 m; Wang et al. (2023)); and *H. brunneoloaurantiacus* occurs in the subalpine belt of Yunnan Province above 3200 m (Wang et al. 2023; this study). This distribution pattern is speculated to be correlated with host plant associations, though further research is needed to confirm this relationship.

##### Hygrophorus brunneoloaurantiacus

Taxon classificationFungiAgaricalesHygrophoraceae

C.Q. Wang, Ming Zhang & T.H. Li, Mycosphere 14: 1776 (2023)

84C62476-FD42-5B37-AE74-5A63FFE7EF8A

901141

Fig. 11A–C, J, M

###### Known distribution.

Yunnan Province, China.

###### Habit and habitat.

Solitary in broadleaf forests, likely associated with *Quercus aquifolioides*; at elevations of 3200–3800 m; fruiting in summer (July–August).

###### Material examined.

China • Yunnan Province: Deqin County (德钦县), Xiaozhongdian Town (小中甸镇), in mixed broadleaf-conifer forests dominated by *Pinus densata*, *Quercus aquifolioides*, *Rhododendron decorum* and *R. parvifolium*, 27°26'24"N, 99°50'21"E, elev. 3280 m, 19 July 2015, *L.P. Tang 1908* (MHKMU TLP-1908), *S.D. Yang 121* (MHKMU YSD-121), *J. Zhao 122* (MHKMU ZJ-122); • Eryuan County (洱源县), Ma’an Mountain (马鞍山), in broadleaf forests dominated by *Quercus aquifolioides* and *Rhododendron* spp., 26°15'21"N, 100°6'4"E, elev. 3630 m, 22 August 2014, *G.L. Zhang 113* (MHKMU ZGL-113); • Yulong County (玉龙县), Yulong Snow Mountain (玉龙雪山), elev. 3230 m, 12 July 2010, *Q. Zhao 689* (HKAS 69410).

**Figure 11.**
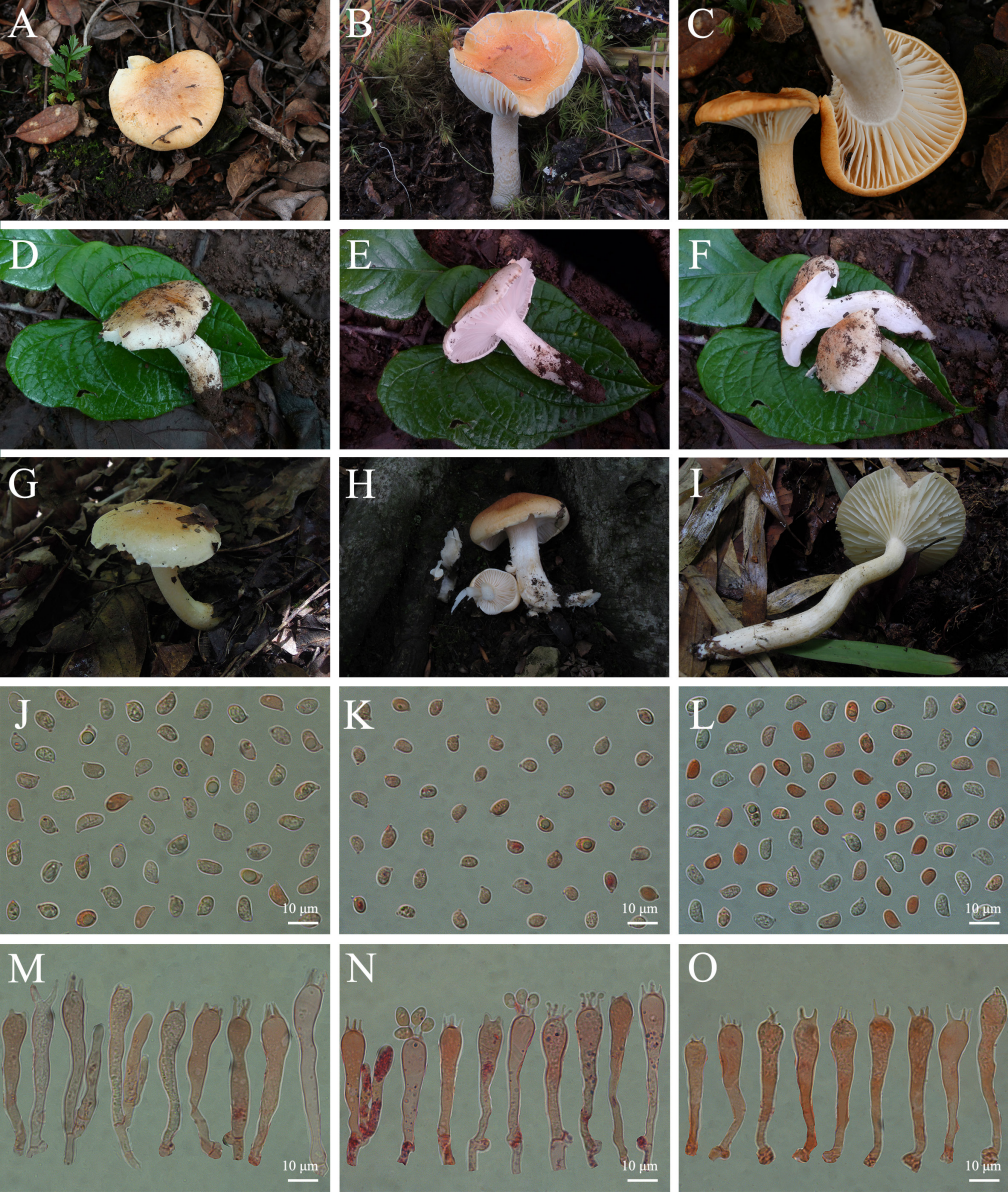
Macroscopic and microscopic features of *Hygrophorus nemoreus* complex. **A–I**. Basidiomata; **J–L**. Basidiospores; **M–O**. Basidia. **A–C, J, M**. *H. brunneoloaurantiacus* (**A, C**. MHKMU ZJ-122; **B**. MHKMU YSD-121); **D–F, K, N**. *H. paulus* (MHKMU TLP-2546, holotype); **G–I, L, O**. *H. shennongjiaensis* (**G**. HKAS 75686; **H**. HKAS 75561; **I**. HKAS 75704, holotype).

###### Notes.

*Hygrophorus brunneoloaurantiacus* is distinguished by a combination of morphological and ecological features. Its basidiomata are medium-sized, with an orangish-yellow to brownish-orange pileus and orangish lamellae that are rarely forked. Microscopically, it produces slender basidia (40–65 × 7–10 μm) and ellipsoid basidiospores measuring 6.5–8 × 4–5.5 μm. Ecologically, this species is considered likely associated with *Quercus aquifolioides* and is currently known only from the subalpine belt (3200–3800 m) of Yunnan Province, south-western China (Wang et al. 2023; this study).

##### Hygrophorus paulus

Taxon classificationFungiAgaricalesHygrophoraceae

L.P. Tang & H.Y. Huang sp. nov.

50C1F8CE-CD3F-55B1-AAD8-1890E40D9584

861811

Fig. 11D–F, K, N

###### Chinese name.

小蜡伞.

###### Diagnosis.

*Hygrophorus paulus* is distinguished from its closest relative, *H. shennongjiaensis*, by its smaller basidiomata, pale orange pileus and small basidiospores (5–7.2 × 4–5 μm). It is currently known only from Yunnan Province, south-western China.

###### Etymology.

The specific epithet “*paulus*” (Latin: small) refers to the notably small basidiomata of this species.

###### Type.

China • Yunnan Province: Qujing City (曲靖市), Junzi Mountain (菌子山), in broadleaf forests dominated by Ericaceae, Fagaceae (*Quercus* spp.) and Juglandaceae, 24°39'1"N, 104°9'51"E, elev. 2270 m, 23 August 2018, *L.P. Tang 2546* (holotype: MHKMU TLP-2546, isotype: HMAS 354463, GenBank Acc. Nos.: ITS = PX868489, nrLSU = PX868389, *tef1-α* = PX907494).

###### Description.

***Basidiomata*** small, firm, fleshy. ***Pileus*** 3 cm diam., broadly convex to plane; pale orange (5A4–5), margin paler; surface dry, covered with fine scales to appressed-fibrillose; context 0.7 cm thick, white (1A1). ***Lamellae*** 0.3 cm wide, decurrent, slightly close, with ca. 50 full lamellae per pileus; lamellulae numerous, narrowed; white (1A1). ***Stipe*** 2.2 cm long, 0.6 cm diam., central, cylindrical; off-white (1A1) to pale orange (5A3); context firm, fibrous, white (1A1). ***Odour*** and ***taste*** unrecorded.

***Basidiospores*** [40/1/1] 5–7.2 × 4–5 μm, Q = (1.11–) 1.16–1.55, Q_m_ = 1.35 ± 0.11, ovoid to broadly ellipsoid or ellipsoid; smooth, thin-walled, hyaline, inamyloid. ***Basidia*** 33–58 × 7–10 μm, clavate, slender, thin-walled, predominantly 4-sterigmate, occasionally 2-sterigmate; sterigmata up to 6 μm long. ***Hymenophoral trama*** slightly divergent, non-gelatinised, of cylindrical hyphae 3–12 μm diam. and inflated elements up to 18 μm diam., thin-walled, hyaline. ***Subhymenium*** next to hymenium, not gelatinised, of short cylindrical to irregular elements 6–55 × 2–5 μm, thin-walled, hyaline. ***Pileipellis*** a trichoderm, of narrow, interwoven hyphae 3–5 μm diam., thin-walled, branched or unbranched. ***Pileal trama*** of dense, thin-walled hyphae 3–19 μm diam. ***Stipitipellis*** a cutis, of hyphae 3–6 μm diam., thin-walled. ***Stipe trama*** of parallel, thin-walled hyphae 4–20 μm diam. ***Clamp connections*** present in all tissues.

###### Known distribution.

Known only from Yunnan Province, China.

###### Habit and habitat.

Solitary in broadleaf forests, likely associated with *Quercus*, at elevations of 2200–2300 m; fruiting in summer (August).

###### Notes.

Phylogenetically, *H. paulus* is sister to *H. shennongjiaensis*. The two species can be distinguished by several morphological and ecological traits: *H. shennongjiaensis* possesses larger basidiomata with a pileus 3–6 cm diam. and a stipe 5–8 × 0.8–1 cm, a darker colouration, slightly larger basidiospores measuring 5.2–8 × 3.8–5 μm and occurs in low-elevation subtropical regions of central and eastern China (Hubei and Zhejiang Provinces).

In addition, *H. paulus* can be distinguished from two morphologically similar taxa. *Hygrophorus brunneoloaurantiacus*, which also occurs in the subalpine belt of Yunnan, differs in having larger basidiomata (pileus up to 15 cm diam., stipe reaching up to 7.5 cm long and 1.5 cm diam.), paler colouration, slightly larger basidiospores (6.5–8 × 4–5.5 μm) and occurrence at higher elevations (> 3200 m) (Wang et al. 2023; this study). *Hygrophorus nemoreus*, a European species, can be distinguished by its darker pileus, shorter and narrower basidia (38–47 × 6–8.5 μm), slightly larger basidiospores measuring 6–8 × 3.8–5.2 μm and scattered to gregarious growth at low elevations (< 1100 m) (Candusso 1997).

##### Hygrophorus shennongjiaensis

Taxon classificationFungiAgaricalesHygrophoraceae

L.P. Tang & H.Y. Huang sp. nov.

58CE32DC-9227-53EC-83A4-0DC1BF5CD296

861812

Fig. 11G–I, L, O

###### Chinese name.

神农架蜡伞.

###### Diagnosis.

*Hygrophorus shennongjiaensis* differs from its closest relative, *H. paulus*, by its larger basidiomata, darker coloration, slightly larger basidiospores (5.2–8 × 3.8–5 μm), and occurrence in low-elevation subtropical areas of central and eastern China (Hubei and Zhejiang Provinces).

###### Etymology.

Latin “*shennongjiaensis*” refers to the type locality, Shennongjia Forest District, Hubei Province, China.

###### Type.

China • Hubei Province: Yichang City (宜昌市), Shennongjia Forest District (神农架林区), Muyu Town (木鱼镇), in bamboo forests, 31°29'23"N, 110°21'33"E, elev. 1800 m, 16 July 2012, *Liu 92* (holotype: HKAS 75704, isotype: MHKMU HHY-1236, GenBank Acc. Nos.: ITS = PX868511, nrLSU = PX868410, *tef1-α* = PX907505).

###### Description.

***Basidiomata*** small to medium-sized, firm, fleshy. ***Pileus*** 3–6 cm diam., broadly convex to plane with a broad umbo; orange to orangish-yellow (5A7–8) or orangish-brown (5B6–7), margin paler (5A4–5); surface viscid when moist, covered with fine scales to appressed-fibrillose; context up to 0.3 cm thick, white (1A1) to cream (1A2). ***Lamellae*** up to 0.4 cm wide, decurrent; lamellulae numerous, narrowed; off-white to cream-white (1A1). ***Stipe*** 5–8 cm long, 0.8–1 cm diam., central, cylindrical; surface off-white (1A1), covered with fine white scales to appressed-fibrillose; context firm and fibrous, white (1A1). ***Odour*** and ***taste*** unrecorded.

***Basidiospores*** [73/3/3] 5.2–8 (–8.5) × 3.8–5 μm, Q = (1.16–) 1.25–1.79 (–2.13), Q_m_ = 1.51 ± 0.17, ellipsoid to long ellipsoid, smooth, thin-walled, hyaline, inamyloid. ***Basidia*** 40–59 × 8.5–10 μm, clavate, slender, thin-walled, predominantly 4-sterigmate, occasionally 2-sterigmate; sterigmata up to 8 μm long. ***Hymenophoral trama*** slightly divergent, non-gelatinised, composed of cylindrical hyphae 3–17 μm diam. and inflated elements up to 25 μm diam., thin-walled, hyaline. ***Subhymenium*** next to hymenium, non-gelatinised, composed of short cylindrical to irregular elements 7–45 × 2–5 μm, thin-walled, hyaline. ***Pileipellis*** an ixotrichoderm to trichoderm, slightly gelatinised, of narrow, closely interwoven hyphae 2.5–5 μm diam., thin-walled, often brownish-coloured by intracellular pigment, branched or unbranched. ***Pileal trama*** of dense, thin-walled hyphae 5–22 μm diam. ***Stipitipellis*** an ixocutis, hyphae 3–5 μm diam., thin-walled. ***Stipititrama*** of parallel, thin-walled hyphae 3–16 μm diam. ***Clamp connections*** present in all tissues.

###### Known distribution.

Hubei (data from this study) and Zhejiang Provinces (GenBank Acc. No. JQ991740), China.

###### Habit and habitat.

Solitary to scattered in broadleaf forests, likely associated with bamboo or *Quercus*; at an elevation of ca. 1800 m; fruiting in summer (July).

###### Additional material examined.

China • Hubei Province: Yichang City (宜昌市), Shennongjia Forest District (神农架林区), Muyu Town (木鱼镇), in bamboo forests, 31°29'23"N, 110°21'33"E, elev. 1800 m, 14 July 2012, *Liu 75* (HKAS 75686); • *ibid*., in broadleaf forests dominated by *Quercus*, 16 July 2012, *Q. Cai 807* (paratype: HKAS 75561).

###### Notes.

Morphologically, *H. shennongjiaensis* is most similar to *H. nemoreus*. However, the latter — a European species — differs in its darker pileus, shorter and narrower basidia (38–47 × 6–8.5 μm) and scattered to gregarious occurrence at low elevations (< 1100 m) (Candusso 1997). *Hygrophorus shennongjiaensis* also bears resemblance to *H. brunneoloaurantiacus* and *H. paulus*. *Hygrophorus brunneoloaurantiacus* is distinguished by its larger basidiomata (pileus up to 15 cm diam., stipe reaching up to 7.5 cm long and 1.5 cm diam.), plane to uplifted, slightly wavy pileus, dark orange colouration and distribution restricted to the subalpine belt (3200–3800 m) of Yunnan Province, south-western China (Wang et al. 2023; this study). A detailed comparison with *H. paulus* is provided in the notes for that species.

#### *Hygrophorus purpurascens* complex

**Notes**. *Hygrophorus purpurascens* complex forms a well-supported monophyletic group. It includes several Chinese taxa: *H. fragilipurpurascens* C.Q. Wang et al., *H. orientipurpurascens* C.Q. Wang et al., *H. pseudopurpurascens* C.Q. Wang et al. and an undescribed lineage (voucher HMAS 280470) as well as the broadly distributed *H. purpurascens* (Alb. & Schwein.) Fr. (Europe–North America) and a North American record labelled H. cf. purpurascens.

Species within the *H. purpurascens* complex are morphologically similar to those of the *H. russula* complex and can be confused in the field. A reliable diagnostic feature distinguishing them is the presence of a cortina-like partial veil in young basidiomata of the *H. purpurascens* complex, a feature absent in the *H. russula* complex (Candusso 1997; Wang et al. 2023; this study).

Within this complex, the three Chinese species — *H. fragilipurpurascens*, *H. orientipurpurascens* and *H. pseudopurpurascens* — share similar morphology and all occur in south-western China, making field identification challenging. They can be distinguished by a combination of altitude distribution, host preferences and subtle morphological characters. Amongst them, *H. pseudopurpurascens* has the broadest altitude range (2400–3900 m) and is the most frequently encountered species (Wang et al. 2023; this study). In contrast, *H. fragilipurpurascens* and *H. orientipurpurascens* are restricted to higher elevations (*H. fragilipurpurascens*: 3100–3300 m; *H. orientipurpurascens*: ca. 3500 m) and are relatively uncommon (Wang et al. 2023; this study). Additionally, *H. orientipurpurascens* possesses denser lamellae (88–108 full lamellae per pileus) compared to the other two species (Wang et al. 2023; this study).

##### Hygrophorus fragilipurpurascens

Taxon classificationFungiAgaricalesHygrophoraceae

C.Q. Wang, Ming Zhang & T.H. Li, Mycosphere 14: 1783 (2023)

4D9BD525-0FC5-5373-BC98-F01677E9EA66

901145

Fig. 12A–C, G–I

###### Known distribution.

Sichuan and Yunnan Provinces, China.

###### Habit and habitat.

Solitary or scattered in mixed broadleaf-conifer forests, likely associated with *Pinus yunnanensis*; at elevations of 3100–3300 m; fruiting from late summer to autumn (August–October).

###### Material examined.

China • Yunnan Province: Lijiang City (丽江市), Lijiang Alpine Botanical Garden (丽江高山植物园), in mixed broadleaf-conifer forests, dominated by *Pinus yunnanensis*, *Quercus aquifolioides* and *Rhododendron concinnum*, 26°58'20"N, 100°10'46"E, elev. 3170 m, 22 August 2020, *T. Huang 472* (MHKMU HT-472); • Shangri-La City (香格里拉市), Radar Station (雷达站), elev. unknown, 4 September 2009, *X.F. Shi 254* (HKAS 71779); • Weixi County (维西县), Qizong Town (其宗乡), elev. 3295 m, 14 October 2011, *X.H. Wang HBB2011-W30* (HKAS 72568).

###### Notes.

*Hygrophorus fragilipurpurascens* is distinguished from other species in the *H. purpurascens* complex by a combination of macro- and micro-morphological characters, as well as its ecological preferences. Its basidiomata are relatively slender, with a pileus 6.5–9 cm diam. and a stipe reaching up to 9 cm long and 1.5 cm diam. The pileus is dark pink to reddish-brown and the lamellae are close (55–80 full lamellae per pileus). Microscopically, it produces ovoid to ellipsoid basidiospores measuring 7–8 × 4.5–5.5 μm (Q = 1.38–1.6).

**Figure 12.**
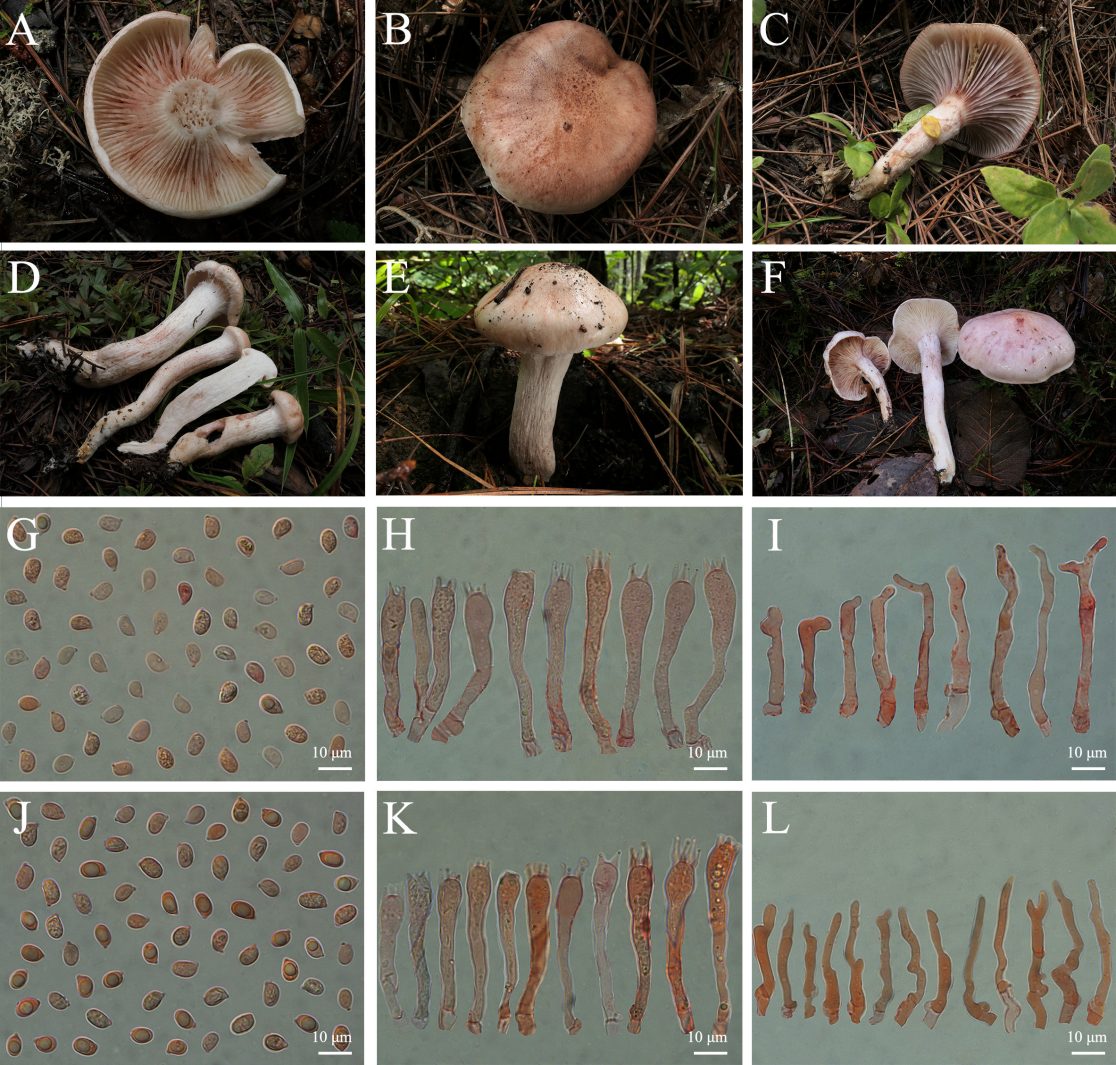
Macroscopic and microscopic features of *Hygrophorus purpurascens* complex. **A–F**. Basidiomata; **G, J** Basidiospores; **H, K**. Basidia; **I, L**. Cystidioid elements on the lamellar edges. **A–C, G–I**. *H. fragilipurpurascens* (MHKMU HT-472); **D–F, J–L**. *H. pseudopurpurascens* (**D**. MHKMU HHY-842; **E**. MHKMU MM-735; **F**. MHKMU NX-164).

An unusual micro-morphological feature observed in our study is the presence of cystidioid elements on the lamellar edges. These measure 30–54 × 3–6 μm, are thin-walled, hyaline, scattered and have apically pyriform, flexuous, irregularly or narrowly clavate shapes, occasionally with branching (Fig. 12I).

Ecologically, this species occurs from solitary to gregarious in the subalpine belt (3100–3300 m), likely forming associations with *Pinus* (Wang et al. 2023; this study). Originally described from Sichuan, its known distribution is here extended to Yunnan Province, south-western China.

##### Hygrophorus pseudopurpurascens

Taxon classificationFungiAgaricalesHygrophoraceae

C.Q. Wang, Ming Zhang & T.H. Li, Mycosphere 14: 1790 (2023)

593B5C84-222D-5708-B993-89194BA16C46

901149

Fig. 12D–F, J–L

###### Known distribution.

Yunnan Province, China.

###### Habit and habitat.

Scattered to gregarious in mixed broadleaf-conifer forests, frequently under *Pinus yunnanensis*; at elevations of 2400–3900 m; fruiting from late summer to autumn (July–October).

###### Material examined.

China • Yunnan Province: Jianchuan County (剑川县), Laojunshan Town (老君山镇), elev. 2418 m, 3 October 2018, *H.Y. Huang 181* (MHKMU HHY-181), *H.Y. Huang 182* (MHKMU HHY-182), *H.Y. Huang 183* (MHKMU HHY-183), *H.Y. Huang 184* (MHKMU HHY-184); • Lijiang City (丽江市), Lijiang Alpine Botanical Garden (丽江高山植物园), in mixed broadleaf-conifer forests, mainly *Pinus yunnanensis*, *Quercus aquifolioides*, *Rhododendron concinnum* and *Vaccinium fragile*, 26°58'26"N, 100°10'46"E, elev. 3100–3200 m, 22 August 2020, *H.Y. Huang 842* (MHKMU HHY-842), *T. Huang 463* (MHKMU HT-463), *M. Mu 735* (MHKMU MM-735), *X. Na 164* (MHKMU NX-164), *Y.J. Pu 356* (MHKMU PYJ-356), *Y.J. Pu 357* (MHKMU PYJ-357), *L.P. Tang 3348* (MHKMU TLP-3348), *W.H. Zhang 473* (MHKMU ZWH-473); • *ibid*., 23 August 2020, *H.Y. Huang 868* (MHKMU HHY-868), *T. Huang 476* (MHKMU HT-476), *M. Mu 755* (MHKMU MM-755), *M. Mu 758* (MHKMU MM-758), *L.P. Tang 3368* (MHKMU TLP-3368); • Lijiang City (丽江市), Xiangshan Free Market (象山农贸市场), elev. unknown, 1 August 2011, *T. Guo 279* (HKAS 71175); • Shangri-La City (香格里拉市), Jiligu (吉利古), 27°44'38"N, 99°57'11"E, elev. 3530 m, 3 August 2021, *T. Huang 680* (MHKMU HT-680); • Shangri-La City (香格里拉市), elev. unknown, 27 July 2006, *Z.W. Ge 1107* (HKAS 50697).

###### Notes.

*Hygrophorus pseudopurpurascens* is marketed as an edible mushroom in Yunnan Province. This species is distinguished by its robust basidiomata, with a pileus up to 13 cm diam. and a stipe reaching up to 16 cm long and 2.7 cm diam. The pileus is pinkish-white to pink and the lamellae are close (55–80 full lamellae per pileus). A key diagnostic feature is the presence of a cortina-like partial veil in young specimens. Microscopically, it produces ellipsoid to oblong basidiospores measuring 6–9 × 4–5.5 μm (Q = 1.33–1.8) (Wang et al. 2023; this study).

The lamellar edges bear cystidioid elements, similar to those observed in *H. fragilipurpurascens*. These structures measure 24–38 × 2.5–6 μm, are thin-walled, hyaline, scattered and have pyriform, narrowly clavate, flexuous or irregular apices, occasionally branched (Fig. 12L).

Ecologically, this species commonly occurs scattered to gregarious under *Pinus yunnanensis* in the subalpine belt (2400–3900 m) of Yunnan Province, south-western China (Wang et al. 2023; this study).

#### *Hygrophorus* sect. *Vividi*

##### Hygrophorus aurantiophyllus

Taxon classificationFungiAgaricalesHygrophoraceae

L.P. Tang & H.Y. Huang sp. nov.

569FF1A7-048F-5A30-8DF3-93DF03EFA951

861813

Fig. 13

###### Chinese name.

橘褶蜡伞.

###### Diagnosis.

*Hygrophorus aurantiophyllus* is distinguished from its closest relative, *H. vividus*, by its paler colour, distant lamellae (25–30 full lamellae per pileus), ovoid to ellipsoid basidiospores (6.5–8.5 × 5–6.2 μm) and solitary to scattered habit.

###### Etymology.

The specific epithet “*aurantiophyllus*” (Latin: “aurantio-”) refers to the distinctive peachy-orange colour of the lamellae in this species.

###### Type.

China • Yunnan Province: Jianchuan County (剑川县), Mapingguan Town (马平关镇), in broadleaf forests dominated by *Lithocarpus* sp., *Quercus aquifolioides*, *Rhododendron concinnum* and *R. decorum*, 26°17'54"N, 99°46'19"E, elev. 3020 m, 6 October 2020, *H.Y. Huang 1004* (holotype: MHKMU HHY-1004-1, isotype: HMAS 354464, GenBank Acc. Nos.: ITS = PX868435, nrLSU = PX868337, *tef1-α* = PX907459).

**Figure 13.**
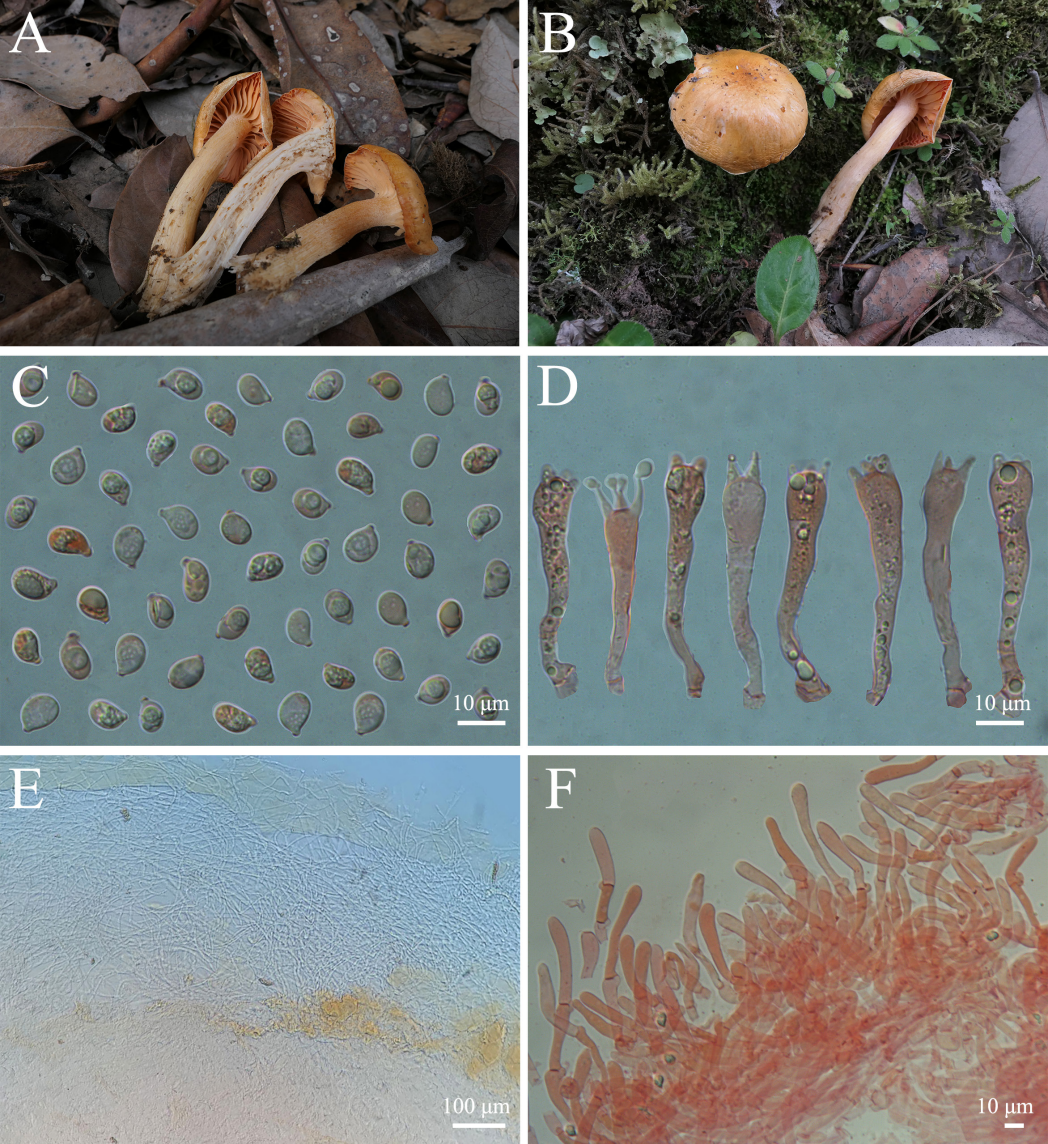
Macroscopic and microscopic features of *Hygrophorus aurantiophyllus* (MHKMU HHY-1004, holotype). **A, B**. Basidiomata; **C**. Basidiospores; **D**. Basidia; **E**. Pileipellis; **F**. Floccule on stipe.

###### Description.

***Basidiomata*** small to medium-sized, firm. ***Pileus*** 3–4 cm diam., broadly convex to plane; orange (5A6) to yellowish-orange (6A6–7); surface dry and smooth; context thin, pale orange (6A2). ***Lamellae*** up to 0.4 cm wide, decurrent, distant, 25–30 full lamellae per pileus; lamellulae numerous, narrowed; peachy-orange (6A5–6). ***Stipe*** 4–5 cm long, 0.9–1 cm diam., central, cylindrical; surface light orange (6A3–4), covered with fine orange scales to appressed-fibrillose; context firm, fibrous, and pale orange (6A2). ***Odour*** distinctive, similar to *H. cossus* and reminiscent of the scent of goat moth larvae (*Cossus cossus*).

***Basidiospores*** [60/2/1] 6.5–8.5 (–9) × 5–6.2 μm, Q = 1.2–1.6 (–1.7), Q_m_ = 1.39 ± 0.10, ovoid, broadly ellipsoid to ellipsoid, smooth, thin-walled, hyaline, inamyloid. ***Basidia*** 34–52 × 7.5–10 μm, clavate, slender, thin-walled, 4-sterigmate; sterigmata up to 8 μm long. ***Hymenophoral trama*** slightly divergent, non-gelatinised, composed of cylindrical hyphae 4–15 μm diam. and inflated elements up to 28 μm diam., thin-walled, hyaline. ***Subhymenium*** next to hymenium, non-gelatinised, composed of short cylindrical to irregular elements 7–45 × 3–7 μm, thin-walled, hyaline. ***Pileipellis*** an ixotrichoderm, gelatinised; the gelatinous matrix often contains yellowish to orange pigment; narrow hyphae, 3–9 μm diam., thin-walled, interwoven, branched or unbranched. ***Pileal trama*** of dense, thin-walled hyphae 4–15 (–28) μm diam. ***Stipitipellis*** a trichoderm, hyphae 4–10 μm diam., interwoven, thin-walled. ***Floccules* (*at stipe apex*)** of compact, erect, branched hyphae with cylindrical to slender clavate terminal elements 24–61 × 5–11 μm, thin-walled. ***Stipititrama*** composed of parallel, thin-walled hyphae 4–22 μm diam. ***Clamp connections*** present in all tissues.

###### Known distribution.

Yunnan Province, China.

###### Habit and habitat.

Solitary to scattered in broadleaf forests dominated by *Lithocarpus* sp., *Quercus aquifolioides*, *Rhododendron concinnum* and *R. decorum*; elev. ca. 3000 m; fruiting in autumn (October).

###### Notes.

This species is morphologically similar to *H. vividus* C.Q. Wang, Ming Zhang & T.H. Li, both of which are currently known only from Yunnan. However, *H. vividus* can be distinguished by its denser lamellae (40–52 full lamellae per pileus), ellipsoid to elongate spores (7–9.5 × 4.5–6 μm), ixocutis-type pileipellis and stipitipellis and scattered to gregarious occurrence at high elevations (> 3800 m; Wang et al. (2023)). Both species share orangish lamellae, a diagnostic trait for the sect. *Vividi* (Wang et al. 2023; this study).

Phylogenetically, *H. aurantiophyllus* forms a sister clade to an undescribed taxon (voucher XML1910693).

## Discussion

### Species diversity of *Hygrophorus* in China

This study provides a phylogenetic overview of *Hygrophorus*, based on multi-locus data (ITS, nrLSU and *tef1-α*) and describes five new species from China: *H. atropurpureus* and *H. ochraceodiscus* in sect. *Hygrophorus*, *H. paulus* and *H. shennongjiaensis* in sect. *Pudorini* and *H. aurantiophyllus* in sect. *Vividi*. To date, at least 104 species have been reported from China, of which 63 are molecularly confirmed (Table 3); the taxonomic status of the remaining reported taxa requires further verification. In addition to these confirmed species, 15 phylogenetic lineages identified in molecular analyses remain formally undescribed (five from this study, seven reported by Wang et al. (2023) and three based on sequences from GenBank); these are excluded from Table 3. Collectively, these results indicate that the diversity of *Hygrophorus* in China is substantially greater than previously recognised, highlighting the need for continued taxonomic and phylogenetic research in the region.

**Table 3.** Distribution of species of *Hygrophorus* in China.

Taxon	Holotype	Distribution in China	References
* Hygrophorus abieticola*	Europe	Heilongjiang, Sichuan, Yunnan	Larsson and Jacobsson (2014); Wang et al. (2023); this study
* H. agathosmoides*	Canada	Inner Mongolia	Bellanger et al. (2021); Huang et al. (2022b)
* H. alboflavescens*	Pakistan	Hunan, Jilin, Sichuan	Naseer et al. (2019); Wang et al. (2023)
* H. alpinus*	China, Yunnan	Shanxi, Sichuan, Yunnan	Huang et al. (2022a); Wang et al. (2023); Zhang et al. (2024)
* H. annulatus*	China, Sichuan	Sichuan, Tibet, Yunnan	Huang et al. (2022b); Wang et al. (2021, 2023)
* H. armeniacus*	China, Yunnan	Yunnan	Wang et al. (2023)
* H. atrofuscus*	China, Yunnan	Yunnan	Huang et al. (2022b)
* H. atropurpureus*	China, Yunnan	Yunnan	this study
* H. aurantioluteus*	China, Sichuan	Sichuan	Wang et al. (2023)
* H. aurantiophyllus*	China, Yunnan	Yunnan	this study
* H. aurantiosquamosus*	China, Tibet	Qinghai, Sichuan, Tibet, Yunnan	Huang et al. (2021b); Wang et al. (2023)
* H. brunneiceps*	China, Yunnan	Gansu, Sichuan, Tibet, Xinjiang, Yunnan	Huang et al. (2022b); Wang et al. (2023)
* H. brunneodiscus*	China, Hunan	Shanxi, Hunan	Wang et al. (2020); Zhang et al. (2024)
* H. brunneodiscoides*	China, Shanxi	Shanxi	Zhang et al. (2024)
* H. brunneoloaurantiacus*	China, Yunnan	Yunnan	Wang et al. (2023); this study
* H. brunneololamellatus*	China, Sichuan	Sichuan	Wang et al. (2023)
* H. brunneolus*	China, Sichuan	Sichuan	Wang et al. (2023)
* H. brunnescens*	China, Sichuan	Sichuan	Wang et al. (2023)
* H. chuxiongensis*	China, Yunnan	Yunnan	Wang et al. (2023)
* H. cinnamoneus*	China, Sichuan	Sichuan	Wang et al. (2023)
* H. deliciosus*	China, Yunnan	Sichuan, Tibet, Yunnan	Wang and Li (2020)
* H. esculentus*	China, Yunnan	Shanxi, Sichuan, Yunnan	Huang et al. (2022a); Wang et al. (2023); Zhang et al. (2024)
* H. flavoalbus*	China, Yunnan	Guizhou, Yunnan	Wang et al. (2023); this study
* H. fragilipurpurascens*	China, Yunnan	Sichuan, Yunnan	Wang et al. (2023); this study
* H. fuscodiscus*	China, Sichuan	Sichuan	Wang et al. (2023)
* H. fuscopapillatus*	China, Sichuan	Sichuan, Yunnan	Wang et al. (2020); this study
* H. gliocyclus*	Sweden	Shanxi, Sichuan, Tibet, Yunnan	Candusso (1997); He and Yang (2021); Huang et al. (2022a); Zhang et al. (2024)
* H. glutiniceps*	China, Guangdong	Guangdong, Hainan, Hunan, Jiangxi	Wang et al. (2020, 2023); this study
* H. griseodiscus*	China, Sichuan	Sichuan	Wang et al. (2020)
* H. habaensis*	China, Yunnan	Yunnan	Huang et al. (2022b)
* H. hedrychii*	Czech Republic	Heilongjiang, Inner Mongolia, Shanxi, Sichuan	Wang et al. (2020, 2023); Zhang et al. (2024); this study
* H. lucorum*	Slovakia	Inner Mongolia, Jilin, Shanxi	Adamčík et al. (2005); Huang et al. (2022a)
* H. magnisporus*	China, Sichuan	Sichuan	Wang et al. (2023); this study
* H. murinidiscus*	China, Sichuan	Sichuan	Wang et al. (2023)
* H. neoerubescens*	Europe	Sichuan, Tibet	Wang et al. (2023); this study
* H. ochraceodiscus*	China, Yunnan	Yunnan	this study
* H. orientalis*	China, Hubei	Hubei, Inner Mongolia, Jilin, Shaanxi, Shanxi, Yunnan	Huang et al. (2021a); Wang et al. (2023); Zhang et al. (2024); this study
* H. orientimarzuolus*	China, Yunnan	Yunnan	Wang et al. (2023)
* H. orientipurpurascens*	China, Yunnan	Yunnan	Wang et al. (2023)
* H. pallidoaurantiacus*	China, Yunnan	Yunnan	Wang et al. (2023)
* H. pallidoagathosmus*	China, Sichuan	Inner Mongolia, Shanxi, Sichuan	Wang et al. (2023); Zhang et al. (2024)
* H. pallidoflavodiscus*	China, Yunnan	Yunnan	Wang et al. (2023); this study
* H. pallidofulvus*	China, Sichuan	Sichuan	Wang et al. (2023)
* H. parvirussula*	China, Yunnan	Sichuan, Tibet, Yunnan	Huang et al. (2018); Huang et al. (2021a); Wang et al. (2023)
* H. paulus*	China, Yunnan	Yunnan	this study
* H. pictus*	China, Hong Kong	Hong Kong	Berkeley and Curtis (1860); taxonomic status requires confirmation
* H. pinophilus*	Sweden	Sichuan, Yunnan	Bellanger et al. (2021); Huang et al. (2022b); Wang et al. (2023)
* H. pseudodiscoideus*	China, Sichuan	Sichuan, Tibet	Wang et al. (2023)
* H. pseudohypothejus*	China, Yunnan	Yunnan	Huang et al. (2022a)
* H. pseudopurpurascens*	China, Yunnan	Yunnan	Wang et al. (2023); this study
* H. pudorinus*	Europe	Sichuan, Yunnan	Wang et al. (2023); this study
* H. qinggangjun*	China, Yunnan	Yunnan	Huang et al. (2021a)
* H. queletii*	Europe	Inner Mongolia	Wang et al. (2023); this study
* H. roseoviolaceus*	China, Chongqing	Chongqing	Wang et al. (2023)
* H. rutilans*	China, Yunnan	Yunnan	Wang et al. (2023)
* H. shennongjiaensis*	China, Hubei	Hubei, Zhejiang	this study
* H. sichuanensis*	China, Sichuan	Sichuan	Wang et al. (2023)
* H. speciosus*	America	Jilin, Shanxi, Sichuan	Peck (1878); Huang et al. (2022a); Wang et al. (2023); Zhang et al. (2024)
* H. subcapreolarius*	China, Yunnan	Yunnan	Wang et al. (2023)
* H. viridiflavidus*	China, Shanxi	Shanxi	Zhang et al. (2023)
* H. vividus*	China, Yunnan	Yunnan	Wang et al. (2023)
* H. xiangjun*	China, Yunnan	Yunnan	Huang et al. (2022a)
* H. yukishiro*	Japan	Anhui, Henan, Zhejiang	Endo et al. (2018); this study
* H. yunnanensis*	China, Yunnan	Yunnan	Huang et al. (2021a)

### Phylogeny of *Hygrophorus*

Although Wang et al. (2023) proposed sect. *Vividi* as a weakly supported sister lineage to sect. *Aurei*, its subgeneric position remained unresolved. The discovery of *H. aurantiophyllus* in this study provides critical evidence that clarifying this placement. In the concatenated phylogeny, sect. *Vividi* is strongly supported as part of a monophyletic clade that also includes sect. *Discoidei* and sect. *Hygrophorus*. The ITS tree also groups sect. *Vividi* with sect. *Hygrophorus*, albeit with lower support. Morphologically, *H. aurantiophyllus* exhibits a distinctive odour of goat moth larvae (*Cossus cossus*) — a trait considered characteristic of subgen. *Hygrophorus* (Lodge et al. 2014; this study). Together, this molecular and morphological evidence strongly suggests that sect. *Vividi* belongs to subgen. *Hygrophorus*.

### Ecological traits of species complexes within *Hygrophorus*

Species complexes are prevalent within the genus *Hygrophorus*, as exemplified by the *H. chrysodon* (Batsch) Fr. complex in sect. *Chrysodontes* (Singer) E. Larss.; the *H. agathosmus* (Fr.) Fr., *H. exiguus* E. Larss., E. Campo & M. Carbone, *H. glutinifer* Fr., *H. olivaceoalbus*, *H. pustulatus* (Pers.) Fr. complex in sect. *Olivaceoumbrini*; the *H. gliocyclus* Fr., *H. hypothejus* (Fr.) Fr. complex in sect. *Aurei*; and the *H. nemoreus*, *H. purpurascens*, *H. russula* complex in sect. *Pudorini* (Lodge et al. 2014; Huang et al. 2018, 2021a, 2021b, 2022a, 2022b; Moreau et al. 2018; Wang and Li 2020; Wang et al. 2020, 2021, 2023; Bellanger et al. 2021). These complexes often exhibit overlapping morphological traits (e.g. size, colouration and odour), making field identification particularly challenging (Lodge et al. 2014; Huang et al. 2018, 2021a, 2021b, 2022a, 2022b; Moreau et al. 2018; Wang and Li 2020; Wang et al. 2020, 2021, 2023; Bellanger et al. 2021). Consequently, ecological characteristics — such as geographic distribution, elevational range, nutritional strategies and host associations — serve as critical tools for delimiting morphologically cryptic species.

#### Distributional patterns

Species complexes display distinct distributional patterns, ranging from cosmopolitan to narrowly endemic. For instance, within the *H. agathosmus* complex, *H. agathosmoides* Lebeuf, E. Larss. & Bellanger exhibits a Holarctic distribution, whereas *H. pinophilus* E. Larss., Sesli & Loizides is restricted to Eurasia and other members show narrow endemism (Bellanger et al. 2021; Huang et al. 2022b; Wang et al. 2023).

#### Host specificity and elevational adaptation

In certain lineages, host specificity is strongly linked to elevational adaptation. This is particularly evident in sect. *Pudorini* (e.g. *H. nemoreus*, *H. purpurascens* and *H. russula* complexes), where species frequently associate with broadleaved trees (Huang et al. 2018, 2021a; Wang and Li 2020; Wang et al. 2023). Their elevational ranges often mirror the altitudinal zonation of their host plants, suggesting co-evolution or ecological niche conservatism.

#### Nutritional mode transitions

Transitions in nutritional mode within complexes are rare, but evolutionarily significant. In the *H. olivaceoalbus* complex, *H. olivaceoalbus* achieves a broad distribution through facultative parasitism, whereas other members (e.g. *H. annulatus*) engage in mutualistic symbiosis and exhibit more restricted ranges (Agerer 2012; Bellanger et al. 2021; Huang et al. 2022b). Notably, *H. olivaceoalbus* displays heightened aggressive behaviour under environmental stress, a strategy that may enhance its dispersal efficiency through adaptive resource exploitation (Agerer 2012).

## Supplementary Material

XML Treatment for Hygrophorus atropurpureus

XML Treatment for Hygrophorus fuscopapillatus

XML Treatment for Hygrophorus glutiniceps

XML Treatment for Hygrophorus hedrychii

XML Treatment for Hygrophorus ochraceodiscus

XML Treatment for Hygrophorus pallidoflavodiscus

XML Treatment for Hygrophorus yukishiro

XML Treatment for Hygrophorus queletii

XML Treatment for Hygrophorus flavoalbus

XML Treatment for Hygrophorus brunneoloaurantiacus

XML Treatment for Hygrophorus paulus

XML Treatment for Hygrophorus shennongjiaensis

XML Treatment for Hygrophorus fragilipurpurascens

XML Treatment for Hygrophorus pseudopurpurascens

XML Treatment for Hygrophorus aurantiophyllus

## Additional information

### Artificial Intelligence (AI) use

The authors accept full responsibility for the content of the manuscript, including the disclosure of any use of AI.

No AI tools were used in the preparation of this manuscript.

### Author ORCIDs

H.-Y. Huang https://orcid.org/0000-0003-4028-3124

W.-H. Zhang https://orcid.org/0000-0002-8983-7346

T.-Z. Liu https://orcid.org/0000-0003-1751-2195

G.-L. Zhang https://orcid.org/0000-0002-9594-0585

S.-D. Yang https://orcid.org/0009-0008-0705-6345

H.-J. Li https://orcid.org/0000-0002-7227-4479

G. Moreno https://orcid.org/0000-0002-9303-7181

L.-P. Tang https://orcid.org/0000-0002-6905-3157

### Data availability

All of the data that support the findings of this study are available in the main text or Suppl. material. materialementary Information.

## Supplementary materials

Supplementary material 1 Phylogenetic tree https://doi.org/10.3897/mycokeys.134.186332.suppl1 Data type pdf Explanation note Phylogenetic tree of *Hygrophorus* based on ITS. RAxML BP values (≥ 70%) and Bayesian posterior probabilities (≥ 0.95) are shown above branches. New species and new phylogenetic species are highlighted in red and blue, respectively. https://binary.pensoft.net/file/1684655This dataset is made available under the Open Database License (http://opendatacommons.org/licenses/odbl/1.0/). The Open Database License (ODbL) is a license agreement intended to allow users to freely share, modify, and use this Dataset while maintaining this same freedom for others, provided that the original source and author(s) are credited.Author: Hong-Yan Huang
