# Supplementary figures and images for: Taxonomic studies on Hygrophorus in China: New species and revised taxonomy
Source: MycoKeys. 2026 Jun 17;134:177–223. doi: 10.3897/mycokeys.134.186332 (PMC13294689; doi:10.3897/mycokeys.134.186332)

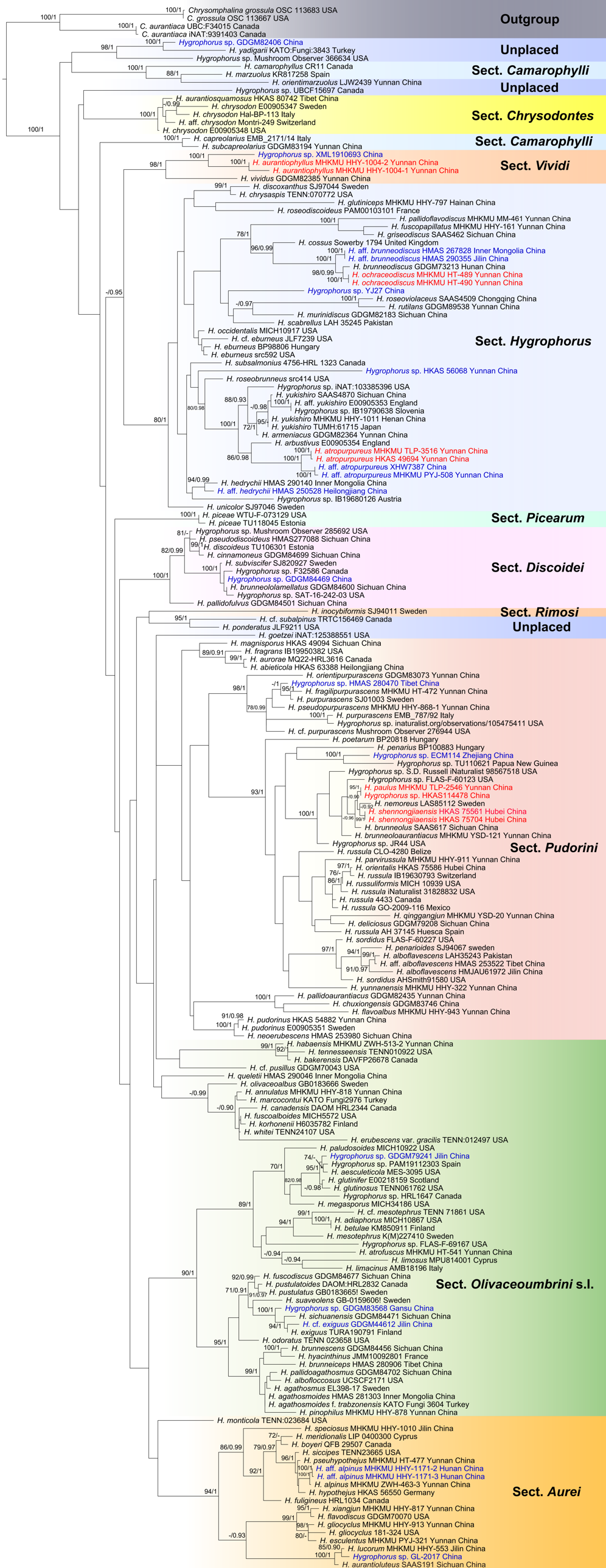

Supplement: Supplementary material 1 — Phylogenetic tree [file mycokeys-134-177-s001.pdf]
